# Randomised controlled trial of SHORT duration antibiotic thERapy for critically ill patients with sepsis (SHORTER trial): protocol for a mixed-methods process evaluation

**DOI:** 10.1136/bmjopen-2026-119173

**Published:** 2026-11-02

**Authors:** Jamie Smith, Helen Mossop, Zoe Walmsley, Phil Mawson, Miranda Morton, Cristina Fernandez-Garcia, Lydia M Emerson, Helen C Hancock, Dawn Teare, Laura Ternent, Ronan McMullan, Timothy S Walsh, Paul Dark, John Simpson, Rashmi Kumar, Margaret Ogden, Thomas P Hellyer, Gosha Colquhoun (formerly Wojcik)

**Affiliations:** 1 School of Health & Social Care, Edinburgh Napier University, Edinburgh, UK; 2 Charite—Universitatsmedizin Berlin, Berlin, Germany; 3 Newcastle University, Newcastle upon Tyne, UK; 4 Population Health Sciences Institute, Newcastle University, Newcastle upon Tyne, UK; 5 Newcastle University Faculty of Medical Sciences, Newcastle upon Tyne, UK; 6 Newcastle Clinical Trials Unit, Newcastle University, Newcastle upon Tyne, Tyne and Wear, UK; 7 Department of Health and Community Sciences, University of Exeter Medical School, Exeter, UK; 8 University of Newcastle upon Tyne, Newcastle upon Tyne, Tyne and Wear, UK; 9 Institute of Health & Society, Newcastle University, Newcastle upon Tyne, UK; 10 Department of Medical Microbiology, Royal Victoria Hospital, Belfast, UK; 11 The University of Edinburgh, Edinburgh, UK; 12 Intensive Care Unit, University of Manchester, Salford, Greater Manchester, UK; 13 Institute of Cellular Medicine, Medical School, Newcastle University Institute of Cellular Medicine, Newcastle upon Tyne, UK; 14 KCL, London, UK; 15 Faculty of Social Sciences, University of Stirling, Stirling, UK; 16 Translational and Clinical Research Institute, Newcastle University, Newcastle upon Tyne, UK; 17 Division of Research Methodology, Wrocław Medical University, Wrocław, Poland

**Keywords:** Sepsis, Antibiotics, Implementation Science, Adult intensive & critical care, Pragmatic Clinical Trial

## Abstract

**Introduction:**

The randomised controlled trial of SHORT duration antibiotic thERapy for critically ill patients with sepsis (SHORTER trial) (ISRCTN40090372) is a multicentre, pragmatic, randomised controlled trial evaluating whether a fixed 5-day course of antibiotics for sepsis in critical care is non-inferior to usual care in terms of mortality, while reducing antibiotic exposure. An embedded process evaluation will examine how organisational, team-based and individual factors shape whether the 5-day protocol is followed as intended (fidelity), whether antibiotic duration actually differs between trial arms (separation) and what drives variation in delivery across approximately 50 UK sites over time.

**Methods and analysis:**

We will undertake a longitudinal mixed-methods process evaluation combining surveys, interviews, observations and routine trial data. Three complementary frameworks guide the analysis at different levels: the Theory of Planned Behaviour (individual clinician decisions), Normalisation Process Theory (team-level embedding of the protocol; assessed using the NoMAD-23 instrument) and the Consolidated Framework for Implementation Research (organisational and system-level conditions). Quantitative components include baseline site profiling, NoMAD-23 survey responses and monthly trial metrics (fidelity, separation, recruitment) across all participating sites. Qualitative components include semistructured interviews and non-participant observations at 10 purposively selected sites. Integration will use site-level case summaries, cross-site typologies and joint displays.

**Ethics and dissemination:**

Favourable ethical opinions were received from Wales REC 4 (REC reference 23/WA/0197) and Scotland A Research Ethics Committee (REC reference 24/SS/0013); Health Research Authority approval is recorded under Integrated Research Application System (IRAS ID) 317788. Findings will be disseminated through peer-reviewed publications, conference presentations, site feedback and accessible summaries for patient/public audiences.

**Trial registration number:**

ISRCTN40090372.

**STRENGTHS AND LIMITATIONS OF THIS STUDY**

- Integrates three complementary theoretical frameworks (Theory of Planned Behaviour, Normalisation Process Theory, Consolidated Framework for Implementation Research) to examine implementation across individual, team and organisational levels, reducing the risk of overlooking explanatory factors at any single level.
- Embedded longitudinal design captures implementation processes in real time rather than relying on retrospective accounts, enabling direct examination of implementation–effectiveness relationships.
- Quantitative and qualitative findings are combined through a staged integration process with prespecified decision rules, making analytic choices transparent and auditable.
- The case-study component covers a purposive subsample of 10 sites; 2- to 3-day observation periods may miss implementation dynamics occurring outside the observation window.
- Usual care may evolve during the open-label trial, potentially reducing separation between arms; this will be measured and explicitly addressed in interpretation.

##  Background

Sepsis is life-threatening organ dysfunction caused by a dysregulated host response to infection.^1^ International guidelines recommend prompt initiation of broad-spectrum antibiotics but offer limited guidance on optimal treatment duration.^2^ In practice, decisions about antibiotic duration integrate clinical factors such as source control, organism identification and susceptibility and biomarker trajectories (including procalcitonin).^2 3^ They are also shaped by microbiology advice, team hierarchy and multidisciplinary influence.^4 5^ Treatment is often prolonged beyond what evidence supports, increasing antibiotic exposure and associated harms.^3^ The global burden of antimicrobial resistance underscores the importance of antibiotic stewardship.^6^ Trials in ventilator-associated pneumonia and complicated intra-abdominal infection have shown that shorter regimens can achieve outcomes comparable to longer courses in selected populations.^7 8^ However, those results do not resolve whether a fixed 5-day regimen is safe across heterogeneous critical-care sepsis populations.

The randomised controlled trial of SHORT duration antibiotic thERapy for critically ill patients with sepsis (SHORTER trial) (ISRCTN40090372) is a National Institute for Health and Care Research Health Technology Assessment (NIHR HTA)-funded (NIHR134101), multicentre, pragmatic, open-label, parallel-group trial testing whether a fixed five×24-hour antibiotic course is non-inferior to usual care for critically ill patients with sepsis.^9^ Adults (≥18 years) admitted to UK critical care units with suspected or confirmed sepsis are randomised, within 4 days of antibiotic initiation, to a fixed five×24-hour index course from the first antibiotic dose (intervention) or to usual care, in which duration is determined by the treating clinical team according to best practice guidance (typically around 9 days). Antibiotic choice, escalation and de-escalation remain at the discretion of the clinical team in both arms. The co-primary outcomes are 28-day all-cause mortality (non-inferiority) and total antibiotic treatment days at 28 days (superiority), with a target sample of 2244 patients across approximately 50 sites. The trial addresses duration rather than antibiotic selection: selection is already governed by local stewardship processes, whereas duration remains weakly evidenced and highly variable, and the two questions are complementary. Implementing a fixed-duration strategy requires behavioural and cultural change across individual, team and organisational levels.^4 10^ Implementation challenges can obscure intervention effects; without understanding delivery and context, pragmatic trial findings are difficult to interpret.^11 12^

This embedded process evaluation will explain how the intervention and trial procedures are delivered in routine intensive care unit (ICU) practice, what shapes fidelity and separation between arms, and how usual care evolves during an open-label trial. We use ‘intervention delivery’ for delivery of the trial protocol to participants (fidelity) and ‘implementation’ for wider adoption, embedding and sustainability in routine practice.

## Objectives

Consistent with Medical Research Council (MRC) guidance,^11^ objectives span three linked domains.

### Primary objectives

Intervention delivery:

1. Quantify fidelity to the fixed 5-day strategy in the intervention arm and the degree of separation between arms over time and by site.
2. Examine stopping decision processes, including clinician confidence, perceived risk and how stopping criteria are interpreted and enacted.

Trial delivery

1. Assess adherence to key trial procedures (screening, eligibility confirmation, consent, randomisation, follow-up, data completeness).
2. Explore how research processes interact with clinical workflows and multidisciplinary decision-making.
3. Determinants and mechanisms.
4. Explore micro (Theory of Planned Behaviour (TPB)), meso (Normalisation Process Theory (NPT)) and macro (Consolidated Framework for Implementation Research (CFIR)) determinants associated with variation in delivery.

### Secondary objectives

1. Usual care dynamics and potential contamination: describe trends in antibiotic duration and decision-making in the usual care arm over time, including evidence of cross-arm diffusion.
2. Local adaptations: describe site-level adaptations to intervention delivery and/or trial procedures, the rationale for adaptations and implications for fidelity and interpretation.

## Theoretical frameworks

Implementing a new antibiotic protocol in the ICU involves change at multiple levels such as individual clinician decisions, team working practices and organisational conditions.^12^ Antibiotic duration decisions are team-based and context-sensitive^4 5^; no single theory can address the full range of determinants in complex and dynamic systems. We therefore use three complementary frameworks, each designed for a distinct level of analysis.

*Individual level*. Why do clinicians stop, or continue, antibiotics beyond day 5? The TPB^13^ examines how attitudes, perceived norms from colleagues and sense of control over stopping decisions shape intentions and behaviour.^14^

*Team level*. How does the 5-day protocol become part of routine multidisciplinary work? NPT^15^ examines the collective work of understanding, engaging with, enacting and appraising a new practice; NPT has also been used in process evaluations of complex healthcare interventions.^16^ The NoMAD-23 instrument will provide an exploratory baseline description of staff expectations about how implementation may work at each site; longitudinal assessment of normalisation will rely on qualitative data (interviews and observations) rather than repeated survey administration.

*Organisational level*. What structural conditions support or hinder implementation across sites? The CFIR^17^ characterises organisational determinants, stewardship infrastructure, resources, leadership and external pressures that may explain variation across National Health Service (NHS) sites.

Each framework is used for its intended level of analysis; we will not infer individual-level constructs from team-level instruments or vice versa. Prespecified constructs and their mapping to data sources are detailed in table 1 and online supplemental S1. Figure 1 summarises the logic model linking site context, implementation activities, hypothesised mechanisms and delivery outcomes.

**Figure 1:**
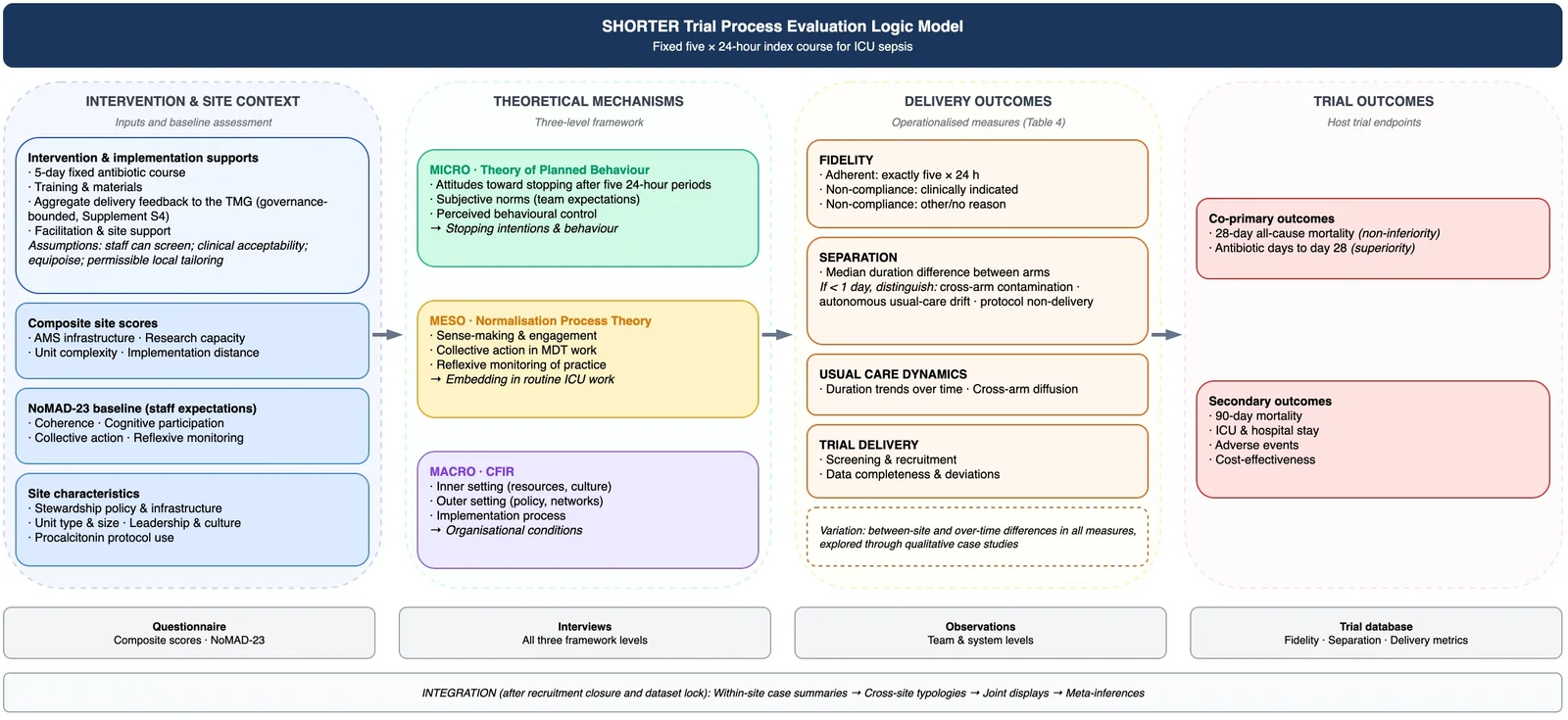
SHORTER trial process evaluation logic model. CFIR, Consolidated Framework for Implementation Research; SHORTER trial, randomised controlled trial of SHORT duration antibiotic thERapy for critically ill patients with sepsis.

**Table 1:** Mapping data types to theoretical frameworks and phases of trial

Data type	Framework(s)	Phase 1 (months 1–12)	Phase 2 (months 13–24)	Phase 3 (months 25–30)
Site questionnaire	CFIR (inner/outer setting)	Baseline	—	—
NoMAD-23 survey	NPT (all constructs)	Baseline	—	—
Documentary analysis	CFIR (context)	Baseline collection	Ongoing review	—
Semistructured interviews	TPB, NPT, CFIR	8–10 per site	As needed	2–4 per site
Non-participant observations	NPT (primarily collective action)	—	2–3 days per site	—
Trial metrics (all sites)	Fidelity, separation, recruitment	Monthly	Monthly	Monthly
Site Principle Investigator (PI) check-in calls	Trial delivery	Monthly	Monthly	As needed

Framework roles: TPB for individual-level determinants (micro); NPT for team-level enactment and embedding (meso); CFIR for organisational context (macro).

CFIR, Consolidated Framework for Implementation Research; NPT, Normalisation Process Theory; TPB, Theory of Planned Behaviour.

## Design and methods

### Overview

This Type 1 effectiveness-implementation hybrid design,^18^ meaning the primary trial outcome remains clinical (mortality non-inferiority) with implementation assessment running alongside, embeds a mixed-methods process evaluation within the SHORTER trial. The process evaluation runs concurrently from site activation through trial completion. Process evaluation data collection began in December 2024, which was Month 1 of the research structure described below and in figure 2. Recruitment to the SHORTER trial began on 30 November 2023; following an extension agreed in early 2026, it is planned to continue until 30 June 2027 (Month 31 of the process evaluation), with trial completion anticipated by 31 May 2028 (Month 42). Figure 2 shows the planned schedule: data collection over Months 1–30, with Phase 2 activities extending across the prolonged recruitment period and Phase 3 spanning the final months of recruitment; integration and synthesis follow recruitment closure and dataset lock (Integration and Synthesis section), with reporting aligned to trial completion. It combines (1) trial-wide quantitative monitoring across all participating sites and (2) in-depth qualitative case studies in a purposive subsample of 10 sites. Data will be collected in parallel and integrated through convergent mixed-methods synthesis.^19^ The evaluation is organised into three phases aligned to trial delivery (early intervention delivery/internal pilot; active recruitment; sustained delivery). Data sources and timing are summarised in table 1 and figure 2. Some UK ICUs employ procalcitonin-guided antibiotic discontinuation protocols, which may provide competing stopping cues that interact with a fixed-duration strategy; site-level biomarker protocol use will be captured through the baseline questionnaire and treated as a contextual variable in cross-site comparison.

**Figure 2:**
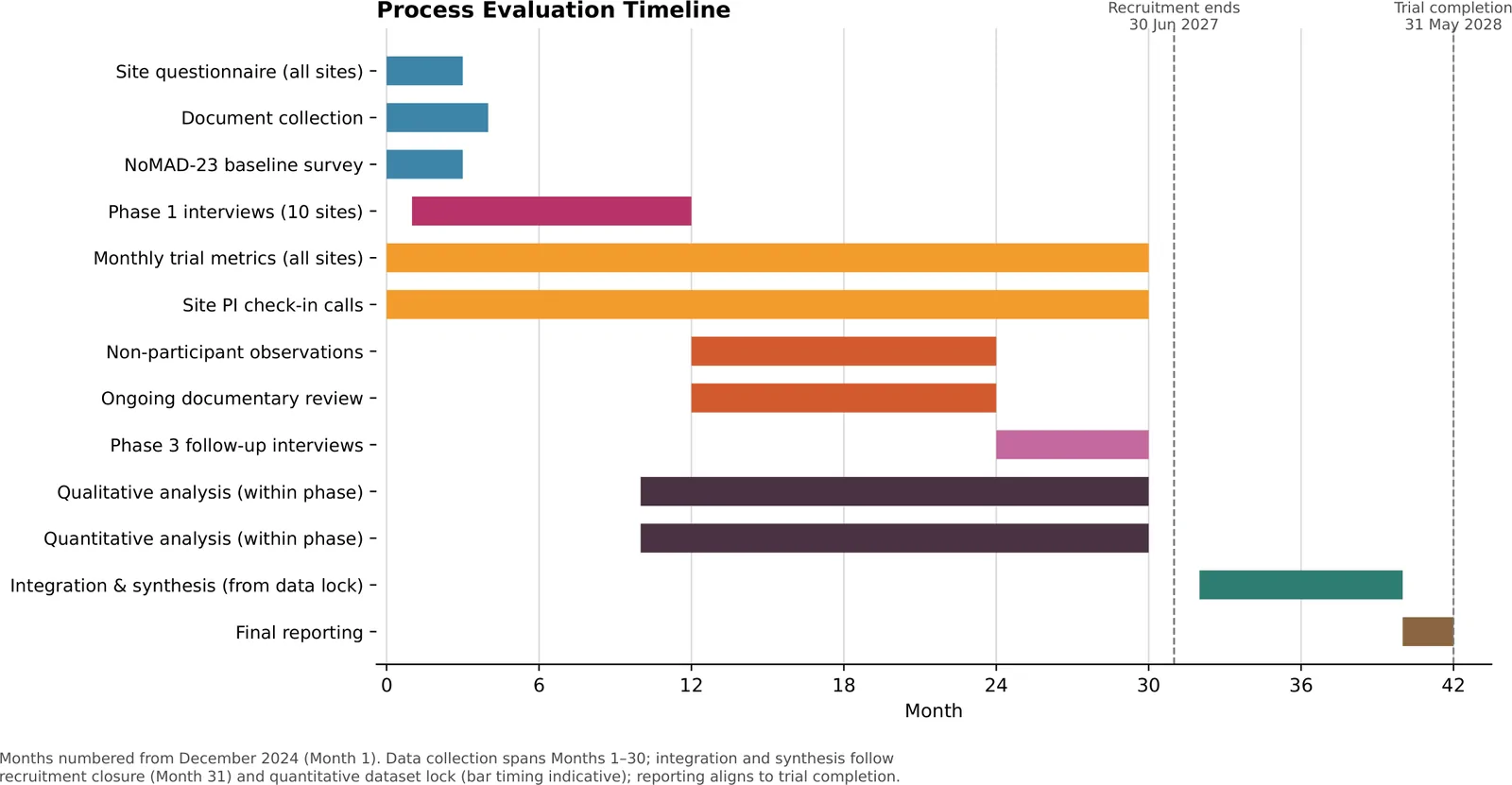
Gantt chart for process evaluation activities.

This protocol was prepared in accordance with the SPIRIT 2025 statement (Standard Protocol Items: Recommendations for Interventional Trials)^20^; the completed checklist, adapted for an embedded process evaluation, is provided as an online supplemental file.

Since the process evaluation runs alongside an open-label pragmatic trial, we must manage the boundary between evaluation and facilitation. During recruitment, feedback to the Trial Management Group will consist of descriptive, aggregate delivery indicators only, and implementation support will follow standard trial monitoring processes. A structured support log, maintained by the Trial Manager and reviewed by the process evaluation team at each analysis phase, will record each contact; aggregate support intensity per site will enter the analysis as a time-varying contextual variable for interpreting within-site changes in fidelity and separation. Full governance arrangements are detailed in online supplemental S4 and summarised in table 2.

**Table 2:** Governance of feedback during recruitment

Feedback type	Shared during recruitment	Timing	Recipient
Aggregate recruitment metrics	Yes	Monthly	Trial Management Group
Aggregate fidelity/separation	Yes	Monthly	Trial Management Group
Data completeness summaries	Yes	Monthly	Trial Management Group
Protocol deviation counts (aggregate)	Yes	Monthly	Trial Management Group
Site-specific interpretive findings	No	Postrecruitment	Final analysis only
Identifiable qualitative data	No	Postrecruitment	Final analysis only
‘Why’ explanations for site performance	No	Postrecruitment	Final analysis only

Support log: Any implementation support provided to sites will be documented (what/when/why) and used to contextualise temporal changes in implementation outcomes during final analysis.

### Sampling strategy

#### Site selection

We will purposively sample 10 sites from the approximately 50 participating sites, balancing depth with breadth and guided by information power.^21^ Sites will be selected using a maximum variation matrix considering: geographic location across UK nations; unit size (tertiles of ICU bed numbers); hospital type (teaching, district general, tertiary/quaternary); existing stewardship infrastructure (assessed through pretrial questionnaire) and research team capacity. Where early trial metrics are available, we will also ensure variation in implementation performance. The Trial Management Group will confirm practical feasibility but will not determine selection based on perceived performance. If selected sites withdraw, replacements will be identified using the same criteria.

#### Participant selection

Within each site, we will recruit participants across professional groups involved in antibiotic decisions and trial delivery (site PI, ICU consultants, pharmacists, microbiologists, research nurses/practitioners, junior doctors, bedside nurses). We anticipate 8–12 unique participants per site (~80–120 total); re-interviews across phases mean interview episodes will exceed participant numbers. Sample adequacy will be appraised prospectively using information power^21^ rather than thematic saturation, which fits poorly with a deductive, framework-driven design; the basis on which adequacy was judged will be documented and reported. Key informants will be re-interviewed across phases to capture longitudinal perspectives; additional participants will be recruited at later phases to capture emerging roles. Participant demographic data including sex, gender, professional role and years of experience will be collected and reported in accordance with Sex and Gender Equity in Research (SAGER) guidelines.^22^ The process evaluation does not exclude participants by sex or gender. As ICU nursing and medical workforces have known gender distributions, demographics will be reported transparently to contextualise findings and analysis will consider whether implementation experiences differ by professional group, seniority or gender. £20 Amazon vouchers are offered for participation in interviews.

### Data collection

#### Instrument development and validation

The NoMAD-23 is a validated instrument for assessing implementation processes based on NPT^23^; the SHORTER-adapted version inserts intervention-specific wording into the standard items without altering item structure, following the developers’ guidance. The adapted items phrase the intervention as prescribing antibiotics for 5 days or fewer; this is the wording presented to respondents, whereas the trial defines adherence as exactly five 24-hour periods from the first antibiotic dose^9^; three general items retain NoMAD’s generic wording about antibiotic practice, and the psychometric properties of the adaptation have not been separately established. The baseline site questionnaire was developed by the research team, informed by CFIR domains and the antimicrobial stewardship literature, reviewed for face and content validity by multidisciplinary co-investigators (intensive care medicine, microbiology, pharmacy, trial management) and Patient Public Involvement (PPI) co-researchers. Semistructured interview topic guides were developed from the TPB questionnaire manual^14^ and NPT and CFIR constructs (online supplemental table S4), reviewed by PPI co-researchers, and will be piloted in first-round interviews and iteratively refined, with amendments documented in the audit trail. The adapted NoMAD-23 and the site questionnaire are provided as administered in online supplemental S5 (NoMAD V.1.1; baseline questionnaire V.2.0); the structured observation tool is provided in online supplemental S3.

Note: TPB=Theory of Planned Behaviour; NPT=Normalisation Process Theory; CFIR=Consolidated Framework for Implementation Research. Questions will be adapted for follow-up interviews (Phase 3) to focus on change over time, sustained practices and lessons learnt. The full topic guide is available from the corresponding author.

#### Phase 1: Early intervention delivery (Months 1–12)

Baseline data collection using the site questionnaire and document gathering (local antibiotic guidelines, stewardship policies, training materials). Site visits will include semistructured interviews with 8–10 staff per site (45–60 min; audio-recorded with consent) exploring current antibiotic practices, initial responses to the protocol, anticipated challenges and team decision-making (online supplemental table S4). Trial delivery metrics (screening, recruitment, deviations, data completeness) will be monitored monthly.

#### Phase 2: Active delivery (Months 13–24)

Non-participant observation of clinical teams caring for enrolled patients will be undertaken during visits (2–3 days, within the REC-approved maximum of 5 days on site) to the 10 case-study sites, focusing on ward rounds, multidisciplinary meetings and handovers where antibiotic duration decisions and trial tasks are negotiated; observation windows will prioritise antimicrobial review rounds and periods of active recruitment. At least one observation session per site will include a weekend day or day-night handover period, as stopping decisions may be deferred or delegated outside regular hours; out-of-hours decision-making will also be explored retrospectively through interview accounts. A structured observation tool (online supplemental S3; domains summarised in table 3) will guide data collection toward decision-making processes and implementation work, not quality-of-care audit. We will purposively seek interviews with clinicians who are less engaged with or critical of the trial protocol, identified through site PI nomination and snowball sampling, to counter self-selection and social desirability effects. Observation procedures and safeguards are detailed in online supplemental S2. Because field notes will not record verbatim speech, subtle interactional dynamics, such as ‘prescribing etiquette’ (implicit norms governing prescribing decisions) and informal sanctioning, may be attenuated; this will be mitigated through immediate postobservation recording of paraphrased accounts and triangulation with interview data. Monthly trial metrics (including fidelity and separation) will continue across all sites.

**Table 3:** Structured observation domains and focus areas

Setting	Observation focus	Specific items to document
Ward rounds	Antibiotic duration discussions	Who initiates discussion of antibiotic duration? How is day-of-therapy counted/communicated? Is trial allocation explicitly referenced? What information is used to inform stopping decisions (biomarkers, clinical trajectory, microbiology)? How is uncertainty expressed and managed? Who has final decision-making authority?
	Team dynamics	Which professional groups contribute to antibiotic discussions? Are nursing staff invited to contribute observations? How are dissenting views handled? Is there evidence of ‘prescribing etiquette’ constraining challenge?
	Documentation	How are antibiotic plans documented? Is trial participation visible in records? Are stop/review dates specified?
Multidisciplinary meetings	Stewardship input	Is there dedicated stewardship discussion? How do pharmacists/microbiologists contribute? Are trial patients discussed differently from non-trial patients?
Nursing handovers	Information transfer	Is antibiotic duration mentioned in handover? Do nurses communicate observations relevant to antibiotic decisions? Is trial participation flagged?
Research processes	Integration with clinical work	How do research staff identify eligible patients? When and how is consent/assent sought? How do research and clinical workflows interact? Are there visible tensions or facilitators?
Informal interactions	Corridor conversations	What informal discussions occur about trial patients or the 5-day strategy? How do staff express confidence, doubt or frustration?

Patient-identifiable information will not be recorded; field notes will use generalised role descriptors rather than names, and contextual detail will be suppressed where necessary to reduce disclosure risk.

#### Phase 3: Sustained delivery (Months 25–30)

Follow-up interviews with 2–4 staff per site, prioritising key informants and sites with contrasting trajectories, focusing on sustained changes in practice, adaptations and unintended consequences.

### Analysis plan

#### Qualitative analysis

Qualitative data, interview transcripts, observation field notes and site documents, will be analysed with Framework Analysis,^24^ which allows both structured analysis using prespecified theoretical constructs and identification of unexpected themes produced with the data. The codebook will be organised by level (individual/TPB, team/NPT, organisational/CFIR) with a separate domain for produced themes. Where data segments engage multiple levels (eg, a clinician describing team norms that shape individual stopping decisions), excerpts will be cross-coded to each relevant framework; primary allocation will be determined by the level at which the phenomenon operates, with disagreements resolved through team discussion and documented in the audit trail. Codes will be applied systematically using MAXQDA 24^25^; framework matrices will be constructed per framework and then aligned through joint displays to preserve level-specific analysis while enabling cross-level interpretation. Quality assurance will include double-coding of ~20% of materials by two researchers (JBS, GC), with disagreements resolved through discussion.^26^ Further quality measures will include iterative framework refinement, audit trail maintenance and participant feedback on emerging interpretations. The full qualitative analysis plan is provided in online supplemental S1 and table S2.

#### Quantitative analysis

Baseline site questionnaire data will generate four composite scores (antimicrobial stewardship infrastructure, research infrastructure, unit complexity, implementation distance; 0–10 each; online supplemental table S5 for purposive sampling and cross-site comparison. All items within each domain are equally weighted; the scoring rubric (items, response options, normalisation and handling of missing items) is provided in online supplemental S1 and table S3. Implementation distance captures the gap between current practice (the unit’s reported consensus on standard antibiotic duration) and the trial protocol (5 days). To avoid circularity, this score draws solely on pretrial baseline data and will not be updated with trial-period durations. Implementation distance will be used descriptively and to inform purposive sampling only; it will not enter any model as a predictive covariate. For typology development, high and low implementation distance will be defined by median split across sites.

Implementation performance will be tracked monthly using routine trial data. The intervention is a fixed five×24-hour index course from the first antibiotic dose; depending on start time, the stop date/time may fall on trial day 6.^9^ Additional antibiotics may be prescribed if clinically indicated, but receipt of more or fewer than 5 days is recorded as non-compliance, with the reason documented. Fidelity will therefore be classified as adherence (exactly five 24-hour periods) or non-compliance; for analysis, documented reasons for non-compliance will be coded as clinically indicated versus other or undocumented. Both Intention To Treat (ITT) and evaluable denominators will be reported, the latter excluding patients who die before adherence to the five×24-hour index course can be assessed.

Fidelity categories will be assigned centrally by the process evaluation team using a written coding manual based on the trial’s prespecified course definition and non-compliance rules, blind to site identity and clinical outcomes. Free-text rationales recorded in the trial dataset will be coded against the manual; a random 10% sample will be independently double-coded (JBS, GC), with disagreements resolved by discussion and consensus and referred to the Chief Investigator where clinical judgement is required. Calibration exercises will precede each analysis phase. Operational definitions of fidelity, separation and variation are consolidated in table 4.

**Table 4:** Operational definitions of fidelity, separation and variation

Term	Operational definition	Data source
Fidelity—adherence	The intervention is a fixed five×24-hour index course from the first antibiotic dose; depending on start time, the stop date/time may fall on trial day 6.^9^ Adherence requires exactly five 24-hour periods.	Routine trial dataset (monthly)
Fidelity—non-compliance: clinically indicated reason	Receipt of more or fewer than five 24-hour periods is recorded as non-compliance. Cases with a documented clinically indicated reason, such as a decision to prescribe additional antibiotics, are coded separately for analysis. Free-text reasons are coded centrally against a written coding manual; a 10% sample is double-coded (JBS, GC).	Routine trial dataset; free-text rationale fields
Fidelity—non-compliance: other/no documented reason	Receipt of more or fewer than five 24-hour periods is recorded as non-compliance. Cases with another documented reason or no documented reason are coded separately for analysis.	Routine trial dataset; free-text rationale fields
Denominators	Fidelity is reported against both intention-to-treat and evaluable denominators; the evaluable denominator excludes patients who die before adherence to the five×24-hour index course can be assessed.	Routine trial dataset
Separation	Absolute difference in median antibiotic duration (calendar days, first to last dose, index episode) between trial arms; supplemented by the difference between arms in the IQR of duration, proportions in each arm exceeding 7 and 14 days, and duration distribution plots. Pragmatic sensitivity threshold: at least 2 days.	Routine trial dataset (monthly)
Variation (in delivery)	Between-site, and within-site-over-time, differences in fidelity, separation and local adaptations to intervention delivery or trial procedures, described by month and trial phase and explored through qualitative case studies.	Routine trial dataset; interviews; observations; documents
Implementation distance	Baseline composite score (0–10; equally weighted items, online supplemental table S5) capturing the gap between current site practice and the 5-day protocol: unit consensus on standard antibiotic duration; presence of an antibiotic prescribing/de-escalation protocol; unit consensus on de-escalation practice. Descriptive and sampling use only; not updated with trial-period data and not used as a predictive covariate.	Baseline site questionnaire (online supplemental table S5)

Antibiotic duration will be measured as calendar days of systemic antibiotic therapy for the index sepsis episode, from first dose to last dose. Overlapping courses (antibiotic switch or escalation) count as continuous therapy; new courses for intercurrent infections distinct from the index episode will be excluded, classified per the trial’s prespecified adjudication rules. Duration measurement includes any post-ICU or postdischarge oral antibiotics where captured by the trial dataset.

Separation will be summarised as the absolute difference in median antibiotic duration between arms, supplemented by the difference between arms in the IQR of duration, the proportion of patients in each arm exceeding 7 and 14 days, and duration distribution plots. If median separation falls below 1 day, qualitative data will be used to distinguish between cross-arm contamination (usual-care clinicians adopting shorter courses), autonomous usual-care drift towards shorter prescribing and protocol non-delivery in the intervention arm. Temporal change in usual-care duration will be visualised using site-level run charts of monthly median duration with trial-phase segmentation, annotated with the timing of relevant local policy changes. Unit-level changes in antibiotic policy during the trial (eg, introduction of local short-course guidance) will be captured through document review at each case-study visit and a brief annual document request to all sites, and incorporated into site case summaries and joint displays. Recruitment and trial delivery metrics (screening rates, data completeness, protocol deviations) will be monitored across all sites.

The NoMAD-23 survey will be administered once, at baseline, for an exploratory, descriptive assessment of staff expectations about how implementation may work. The SHORTER-adapted wording has not been separately validated, and the survey is not used as a longitudinal measure. A single baseline administration limits respondent burden on a workforce with high survey exposure, and repeated administration risks progressively unrepresentative responses as engaged staff self-select; change in implementation over time will instead be examined through interviews, observations and trial metrics. Targeted follow-up of low-fidelity sites using abbreviated NPT-based questions was considered and rejected because differential measurement across sites would compromise comparability; Phase 3 interviews will instead prioritise sites with contrasting trajectories. Site-level construct means (coherence, cognitive participation, collective action, reflexive monitoring) will generate descriptive baseline profiles. Sites with fewer than five valid responses or a response rate below 30% of eligible staff will be excluded from NoMAD-based comparisons; response-validity and item-completeness rules and the full inclusion hierarchy are prespecified in online supplemental S1, and excluded sites’ normalisation processes will be characterised qualitatively. If baseline NoMAD data prove unusable across multiple sites (eg, widespread low response), we will substitute a brief qualitative baseline assessment using deductive NPT constructs from Phase 1 interview data. Because respondents are reporting expectations before delivery, baseline scores may be optimistic; we will interpret high scores cautiously and triangulate with qualitative accounts of initial expectations from Phase 1 interviews.

Exploratory descriptive analyses will examine two focal associations at the site level (ecological design, N≈50 sites): stewardship infrastructure score and site-level fidelity, and NoMAD collective action score and site-level separation. These site-level associations (N≈50) preclude patient-level mixed models; analyses will use scatter plots with confidence bands, rank-based correlations and graphical summaries. Where site-level cell sizes are small (eg, fewer than 10 intervention-arm patients), we will flag the association and draw on qualitative data to interpret the pattern. CIs will accompany all estimates.

#### Integration and synthesis

We will describe usual care antibiotic duration by site and over time, interpreting separation alongside qualitative accounts of communication, shared learning and stewardship activity. If separation narrows, we will treat this as an implementation finding in its own right and address it directly when interpreting trial outcomes.

Bringing quantitative and qualitative findings together is the central analytical challenge of this evaluation. Integration will proceed as staged synthesis after recruitment closes and the quantitative dataset is locked—not iteratively during delivery—to prevent early interpretations from feeding back into how sites are supported. Any process evaluation observations informing Trial Management Group (TMG) communications during recruitment are governed by the aggregate feedback provisions in online supplemental S4. The stages are: (1) within-site case summaries combining baseline context, staff expectations (NoMAD), observed work processes, interview findings and delivery metrics; (2) cross-site typologies of implementation trajectories; (3) joint displays aligning quantitative indicators with qualitative explanations by construct and time phase. For typology construction, ‘high’ and ‘low’ quantitative indicators will be defined using median splits across sites (eg, fidelity above/below the site-level median proportion; separation above/below the median duration difference; NoMAD construct means above/below the site-level median). Median splits serve as pragmatic thresholds for organising joint displays and typology construction, not for hypothesis testing; sensitivity analyses will examine alternative cut points, including quartiles and a pragmatic sensitivity threshold of at least 2 days, selected relative to the anticipated contrast between the fixed 5 day course and usual-care durations of approximately 9 days assumed in the host trial design. Where qualitative evidence conflicts with quantitative classification (eg, staff report strong engagement but measured fidelity is low), we will preserve and analyse the mismatch as potentially informative implementation findings, rather than resolve it by privileging one data type.

Integration will also address heterogeneity by infection type. Clinical equipoise and prescribing behaviour are likely to vary across infection categories (eg, pneumonia, bloodstream, intra-abdominal); where sample size permits, we will explore infection-type subgroup patterns descriptively alongside qualitative accounts of how infection source shapes stopping decisions.

### Data management

All data will be managed in accordance with Newcastle upon Tyne Hospitals NHS Foundation Trust policy. Qualitative data will be transcribed by an approved service with confidentiality agreements, pseudonymised using unique identifiers and stored on secure servers with restricted access. Quantitative process data will be held in the trial’s managed data systems with documented entry and consistency checks; coding decisions and data transformations will be recorded in the version-controlled analysis log (online supplemental S1).

### Reflexivity

The multidisciplinary team brings together complementary expertise in implementation science, critical care nursing and health services research. Interviews and observations will be conducted by JBS, a Senior Research Fellow, registered nurse and experienced mixed-methods health services researcher with extensive clinical and applied research experience, and GC, an experienced research methodologist specialising in qualitative methods and implementation research; neither is employed by participating sites nor involved in trial delivery decisions. Regular debriefs and reflexive journals will document methodological decisions, assumptions and potential biases. We will document process evaluation contacts and trial-support actions over time and consider these when interpreting temporal changes in fidelity and separation. A recognised limitation of TPB is the gap between intention and behaviour: clinicians may express willingness to stop antibiotics at day 5 yet act differently under time pressure, clinical uncertainty or competing team norms. The process evaluation addresses this through structured observations of prescribing practice during ward rounds and handovers, and by triangulating self-reported TPB measures with behavioural data from the trial dataset—specifically, actual antibiotic duration in the intervention arm.

### Patient and public involvement

Two PPI co-researchers (RK, MO) are co-applicants on the SHORTER grant and members of the Trial Management Group, involved from the grant application stage onwards. They contributed to the research questions and selection of outcome domains, to study design and to the development of participant-facing materials and interview topic guides, including their acceptability and burden for participants; both reviewed this manuscript prior to submission. PPI contributors are not involved in recruiting staff participants. During analysis, they will participate in interpretation workshops to ensure emerging findings and implementation recommendations reflect patient and family priorities, and they will contribute to accessible summaries for dissemination. Independent PPI representation on the Trial Steering Committee provides ongoing oversight.

## Ethics and dissemination

Favourable ethical opinions were received from Wales REC 4 (REC reference 23/WA/0197) and Scotland A Research Ethics Committee (REC reference 24/SS/0013); Health Research Authority approval is recorded under IRAS ID 317788. The SHORTER trial is registered (ISRCTN40090372) and sponsored by Newcastle upon Tyne Hospitals NHS Foundation Trust. Staff recruited for interviews and the observation component will provide written informed consent; survey completion after receipt of study information will be taken as implied consent. Observations will focus on work processes, not patient care quality; staff may request observer withdrawal at any time; safeguards are detailed in online supplemental S2. Field notes will record researcher descriptions of work processes, decision-making sequences and contextual conditions. Direct quotations of staff speech will not be recorded during observations, as specified in the approved consent materials (online supplemental S2, S2.8). Reported accounts of observed events will take the form of analytic descriptions. Interview quotations will be attributed using generalised role descriptors, with contextual details suppressed where necessary to reduce disclosure risk. Data will be pseudonymised in accordance with the.^27^ Audio recordings and observation field notes will be destroyed on completion of the study, in line with the approved consent materials (online supplemental S2); pseudonymised interview transcripts will be retained in accordance with the sponsor-approved retention schedule, then securely destroyed.

Findings will be disseminated through peer-reviewed publications alongside the main trial outputs, presentations at critical care and implementation science conferences and accessible summaries for patient/public audiences and clinical/policy networks.

### Trial status

The published host-trial manuscript is based on protocol V.6.0, dated 24 September 2025.^9^ The International Standard Registered Clinical/soCial sTudy Number (ISRCTN) record lists first enrolment on 30 November 2023. Recruitment is planned to continue until 30 June 2027, with trial completion anticipated by 31 May 2028. At the time of this resubmission (September 2026), recruitment is active and Phase 2 process evaluation data collection is under way. ISRCTN registration number: ISRCTN40090372 (registered 12 December 2023).

## Supplementary material

10.1136/bmjopen-2026-119173 online supplemental file 1

10.1136/bmjopen-2026-119173 online supplemental file 2

10.1136/bmjopen-2026-119173 online supplemental file 3

10.1136/bmjopen-2026-119173 online supplemental file 4

10.1136/bmjopen-2026-119173 online supplemental file 5

10.1136/bmjopen-2026-119173 online supplemental file 6

10.1136/bmjopen-2026-119173 online supplemental file 7
