# Supplementary material for: Randomised controlled trial of SHORT duration antibiotic thERapy for critically ill patients with sepsis (SHORTER trial): protocol for a mixed-methods process evaluation
Source: BMJ Open. 2026 Nov 2;16(11):e119173. doi: 10.1136/bmjopen-2026-119173 (PMC13735336; doi:10.1136/bmjopen-2026-119173)
Supplement: Supplementary file 1 [file bmjopen-16-11-s002.pdf]

Supplement S2: Detailed Ethics and Regulatory Procedures for the SHORTER Trial Process Evaluation

S2.1 Ethical Approvals and Governance

Favourable ethical opinions were received from Wales REC 4 (REC reference 23/WA/0197) and Scotland A Research Ethics Committee (REC reference 24/SS/0013); Health Research Authority approval is recorded under IRAS ID 317788. The SHORTER trial is registered on ISRCTN (ISRCTN40090372) and sponsored by Newcastle upon Tyne Hospitals NHS Foundation Trust. All participating sites will obtain local NHS Research & Development approval prior to recruitment. The trial will be conducted in accordance with Good Clinical Practice, the UK Policy Framework for Health and Social Care Research, and relevant capacity legislation in each UK jurisdiction.

S2.2 Consent Procedures

Main trial participants

Given the critical illness context and the likelihood that eligible patients may lack decision-making capacity, consent procedures follow UK legal frameworks and sponsor/REC requirements:

- **England, Wales and Northern Ireland:** Mental Capacity Act frameworks (e.g., Personal or Nominated Consultees)
- **Scotland:** Adults with Incapacity (Scotland) Act 2000 (e.g., Welfare Attorney/Guardian or nearest relative in statutory order)
- If capacity returns, participants will be re-approached and asked to provide informed consent for ongoing participation, with the option to withdraw

Process evaluation participants (staff)

Clinical and research staff participating in interviews or the observation component will provide informed, written consent; survey participation is voluntary, with completion following receipt of study information taken as implied consent. Staff will receive study information in advance (≥24 hours where feasible), participation is voluntary, and consent may be withdrawn at any time without consequence.

S2.3 Clinical Observations

Observations focus on implementation and trial work processes (e.g., how antibiotic duration decisions are made and documented; how trial screening/recruitment tasks are integrated into routine work), not on auditing quality of care.

Procedures

- Unit-level permission will be obtained from the ICU Clinical Lead and Unit managers (or equivalent)
- Staff will be notified in advance (e.g., email and posters) and reminded at the start of shifts
- Staff may opt out or request observer withdrawal at any time
- Researchers will take a non-participant stance and will not contribute to clinical decisions or provide clinical advice
- Observations will prioritise settings where decision-making is visible (e.g., ward rounds, multidisciplinary meetings and handovers)
- Observations will avoid observing intimate personal care

S2.4 Managing Consent in Observations

We acknowledge that in real-world ICU environments, not all individuals present during observations (e.g., visiting clinicians, patients, families) will have given explicit prior consent. Our approach, approved by Research Ethics Committees, mitigates risks by:

- Limiting data capture to staff behaviours and work processes directly relevant to intervention and trial delivery
- Not recording patient-identifiable information
- Providing on-the-spot explanation to any patient/family present during observed interactions and honouring any request for observer withdrawal
- Recording only pseudonymised field notes

S2.5 Safety and Oversight

Participant safety is monitored by an independent Data Monitoring Committee (DMC) and Trial Steering Committee (TSC). The DMC reviews unblinded safety data at pre-specified intervals and may recommend trial modification or termination. Serious adverse events will be reported according to sponsor, NIHR and REC requirements, including expedited reporting where required.

Observation safeguards and escalation pathway

Although the process evaluation is not a quality-of-care audit, researchers may witness events that raise serious and immediate patient-safety concerns. In such instances, researchers will follow a pre-specified escalation pathway:

1. Raise the concern immediately with the responsible senior clinician on shift (or the ICU Clinical Lead/Unit management where appropriate)
2. Inform the site Principal Investigator

3. Document the occurrence in a secure internal log (no patient identifiers)
4. Escalate through sponsor governance channels where required, in line with local policy

S2.6 Data Protection and Confidentiality

Working research datasets will be pseudonymised and stored on secure, access-restricted servers. Audio recordings will be stored securely with restricted access until destruction in accordance with the approved consent materials. Processing will comply with the Data Protection Act 2018 and UK GDPR. Staff participants retain standard rights of access, rectification and withdrawal, subject to legal and governance requirements. Interview transcripts and field notes will be pseudonymised using unique identifiers. Interview quotations will be attributed using generalised role descriptors, with contextual details suppressed where necessary to reduce disclosure risk.

S2.7 Patient and Public Involvement

Two lived experience co-researchers (RK, MO) contributed to the development of this process evaluation protocol and serve on the Trial Management Group. Independent PPI representation is included on the Trial Steering Committee. PPI contributors will be involved in:

- Review of participant-facing materials
- Interpretation of findings
- Development of accessible summaries for public audiences
- Dissemination planning

S2.8 Observation Participant Information Sheet and Consent Form

The participant information sheet and consent form for the observation component are reproduced on the following pages exactly as approved (document 10238-SHORTER-PEPISCF [Observations], version 2.0, 24 September 2025). Site-adaptable fields in the approved master (the local complaints contact, and the information-sheet version and date confirmed at the point of consent) appear as in the master document.

S2.9 Interview Participant Information Sheets and Consent Forms

The corresponding approved documents for interview participation follow (10238-SHORTER-PEPISCF [In-Person], version 3.0, and 10238-SHORTER-PEPISCF [Telephone], version 3.0; both 24 September 2025). Written consent is recorded on these forms; digital voice recordings are destroyed on completion of the study, as stated in the approved materials.

FUNDED BY NIHR | National Institute for Health Research

This study is funded by the National Institute for Health Research (NIHR) Health Technology Assessment (HTA) Programme (16/93/01). The views expressed are those of the author(s) and not necessarily those of the NIHR or the Department of Health and Social Care.

A randomised controlled trial of SHORT duration antibiotic thERapy for critically ill patients with sepsis (SHORTER TRIAL)

PROCESS EVALUATION – INFORMATION SHEET (Observations)

Introduction

You are being invited to contribute to this research study. Before you decide whether or not you would like to take part, it is important for you to understand why the research is being done and what it involves. Please take time to read the following information carefully. Please ask if there is anything that is not clear or if you would like further information. Take as long as you need to decide whether or not you wish to take part.

Study summary

Sepsis is a syndrome of life-threatening organ dysfunction secondary to infection and is a leading cause of death worldwide. A third of patients with sepsis die in hospital and sepsis is estimated to cost the National Health Service (NHS) £15 billion per year. Sepsis is therefore a common reason for admission to a critical care unit, accounting for one-third of general adult critical care admissions, equating to approximately 40,000 patients per year in the UK.

Antibiotics are lifesaving in sepsis and current international and NICE guidelines recommend the initiation of antibiotics within one hour of recognition of suspected sepsis. Patients presenting with life-threatening suspected sepsis have broad-spectrum antibiotics initiated in a ‘hard-and-fast’ approach due to the perceived risk of under-treating a severe infection. Once antibiotics are initiated, the optimal duration is unknown. Previous sepsis guidelines recommended 7-10 days of antibiotics, but updated guidelines encourage shorter duration antibiotics, although this is a weak recommendation based on the very low quality of evidence. UK Health Security Agency (UKHSA) (previously Public Health England) and NICE both promote antibiotic stewardship, encouraging the shortest duration of antibiotics needed and discontinuation of antibiotics if there is no evidence of infection. In this study, we aim to find out if a shorter duration of antibiotics is non-inferior to the usual more than 7-day antibiotic treatment.

We also want to find out nurses’ and doctors’ views on how easy or difficult it was to follow the protocol. This will give us valuable practical information that can be shared with other units. This part of the study is called a ‘process evaluation’. **You are being asked if you are willing to participate in the process evaluation**.

This study is funded by the National Institute for Health Research (NIHR) Health Technology Assessment (HTA) Programme (16/93/01). The views expressed are those of the author(s) and not necessarily those of the NIHR or the Department of Health and Social Care.

What is the purpose of this study?

This study is intended to help us explore the processes involved in delivering the intervention and any factors and mechanisms of their interaction likely to impact the trial outcomes. Our objectives are to establish the extent to which the intervention is implemented as intended; to ascertain how clinical staff understand and respond to the intervention; to identify barriers and facilitators that affect the implementation of the trial; and explore the importance of the context in the uptake of the intervention.

Why have I been invited?

You have been approached because you are part of the clinical and/or research team directly involved in caring for patients who are enrolled in the study. You have had first-hand experience managing patients with the approaches we are comparing.

Do I have to take part?

No, it is up to you to decide whether you would like to take part. If you agree, you will be given this information sheet to keep and be asked to sign a consent form. If you agree, you are free to withdraw at any time without giving a reason.

What will happen if I agree to take part?

The researcher will spend a maximum of 5 days on the unit observing the ICU clinical team and their interactions with each other and the decision-making process related to patients' antibiotic treatment. This will be a passive observation and staff will not be needed to participate. The researcher will work a variable pattern across multiple shifts that will be agreed upon with the Clinical Lead of your unit.

Information will only be collected when the ICU team are communicating with one another at the bedside regarding a patient's antibiotics. From time to time you may see the researcher attending ward rounds, and discussing antibiotic treatment plans with the ICU team to verify that antibiotic changes related to the trial protocol have been initiated as planned. If the researcher sees something of interest, they may approach you and ask you about your decisions related to your antibiotic practice. These discussions will be brief (less than 5 minutes), and the researcher will be careful not to ask any questions in front of patients, relatives or any other clinical staff. It is impossible to predict how long each observation will last as this will depend on clinical situation, staff rotas and the researcher's availability to attend the unit, but it is anticipated that this should not be longer than 3-4 hours during any given shift.

This study is funded by the National Institute for Health Research (NIHR) Health Technology Assessment (HTA) Programme (16/93/01). The views expressed are those of the author(s) and not necessarily those of the NIHR or the Department of Health and Social Care.

The staff members in the ICU clinical team are the focus of this research; no identifiable patient data will be collected or stored at any time. There will no sampling of any type taken from the patient. Paper-baed notes will be taken by the researcher as they observe the clinical team. Observations do not judge the staff's ability to deliver bedside care. However, if unsafe/unprofessional practice is observed, the usual professional procedures will be followed. The notes will be stored in a safe place and only the research team will have access to them. The notes will be destroyed in accordance with University regulations and/or if you should decide to withdraw from the study. You will not be identified by name or any other person-specific details.

The observation will be undertaken by Jamie Smith who is an experienced Research Fellow with an extensive critical care research background. Jamie will ensure patient confidentiality and dignity are maintained at all times.

What are the possible benefits of taking part?

There are no direct benefits to you taking part in this study, but the results from this study might help to improve the healthcare of patients in the future.

What are possible disadvantages of taking part?

There are no known disadvantages to taking part in this study.

What if there are any problems?

If you have a concern about any aspect of this study, please contact: Dr Gosha Colquhoun: G.Colquhoun2@napier.ac.uk or Dr Jamie Smith: J.Smith7@napier.ac.uk who will do their best to answer your questions.

What will happen if I don't want to carry on with the study?

You are free to withdraw your consent to participate in this study **at any time** without giving a reason but we will keep the information we already have.

What happens when the study is finished?

Anonymised field notes will be analysed for themes and the subsequent report will be shared with the trial Chief Investigator, trial management committees and the funder. The results of the research may be published through medical publications. Individual participants will not be identifiable in any published results.

Will taking part in the study be kept confidential?

All the information we collect during the research will be kept confidential and the filed notes will be destroyed upon completion of the study. There are strict laws which safeguard your privacy at every stage. The steps to protect your data are described at the end of this leaflet.

Who is organising and funding the research?

This study is sponsored by the Newcastle upon Tyne Hospitals NHS Foundation Trust. The study is being funded by the National Institute for Healthcare Research – Health Technology Assessment Programme (NIHR HTA).

Who has reviewed the study?

All research in the NHS is looked at by an independent group of people called a Research Ethics Committee. A favourable ethical opinion has been obtained from Wales REC 4 Research Ethics Committee. NHS Management Approval has also been given. Former participants and relatives and the local Patient Advisory Group also helped us design this trial.

Researcher Contact Details

If you have any further questions about the study please contact Dr Gosha Colquhoun: G.Colquhoun2@napier.ac.uk or Dr Jamie Smith: J.Smith7@napier.ac.uk.

Independent Contact Details

If you would like to discuss this study with someone independent of the study, please contact: Dr Aisling McMahon: AislingMcMahon@mater.ie.

Complaints

If you wish to make a complaint about the study, please contact:

<insert contact details> *to be adapted depending on the research site.*

This study is funded by the National Institute for Health Research (NIHR) Health Technology Assessment (HTA) Programme (16/93/01). The views expressed are those of the author(s) and not necessarily those of the NIHR or the Department of Health and Social Care.

Further information on Data Protection arrangements

All the information we collect during the research will be kept confidential and there are strict laws to safeguard your privacy at every stage.

All information will be stored by Edinburgh Napier University and Newcastle University. It would only be transferred to other researchers after all necessary approvals were in place.

In order to monitor and audit the study, we will ask your consent for research representatives from the study sponsors to access data collected during the study, where it is relevant to taking part in this research.

How will we use your information?

We will need to use information from you for this research project. This information will include your:

- Name
- Contact details – work telephone number and email address
- Name of institution
- Position in institution

People will use this information to do the research or to make sure that the research is being done properly.

People who do not need to know who you are will not be able to see your name or contact details. Your data will have a code number instead.

Individuals from the Edinburgh Napier University, Newcastle upon Tyne Hospitals NHS Foundation Trust, Newcastle University and regulatory organisations may listen to recordings of interviews/discussions or read the transcripts to check the accuracy of the research study. Audio-recorded interviews may be transcribed by an external third party and will be stored securely and confidentially. The only people in Edinburgh Napier University and Newcastle University, who will have access to information that identifies you, will be people who will audit the data collection process.

We will keep all information about you safe and secure.

Once we have finished the study, we will keep some of the data so we can check the results. We will write our reports in a way that no one can work out that you took part in the study.

This study is funded by the National Institute for Health Research (NIHR) Health Technology Assessment (HTA) Programme (16/93/01). The views expressed are those of the author(s) and not necessarily those of the NIHR or the Department of Health and Social Care.

Where can you find out more about how your information is used?

You can find out more about how we use your information:

- at www.hra.nhs.uk/information-about-patients/
- from our leaflet available from www.hra.nhs.uk/patientdataandresearch
- by asking one of the research team, or
- by contacting the Data Protection Officer at either:
  - Edinburgh Napier University. Email: dataprotection@napier.ac.uk or call 0131 455 2946
  - NHS Lothian Lothian.DPO@nhs.net or call 0131 465 5444

Thank you for your time

FUNDED BY NIHR | National Institute for Health Research
This study is funded by the National Institute for Health Research (NIHR) Health Technology Assessment (HTA) Programme (16/93/01). The views expressed are those of the author(s) and not necessarily those of the NIHR or the Department of Health and Social Care.

A randomised controlled trial of SHORT duration antibiotic thERapy for critically ill patients with sepsis (SHORTER TRIAL)

PROCESS EVALUATION - CONSENT FORM

Centre Name: ______________________

Please **initial** box

1. I confirm that I have read and understand the Process Evaluation Information Sheet (Version _._, dated _ _ / _ _ / _ _ _ _) for the above study and have had the opportunity to consider the information, ask questions and have these questions answered satisfactorily. ☐
2. I understand that my participation is voluntary and that I am free to withdraw at any time, without having to give a reason for doing so. ☐
3. I understand that relevant sections of my data collected during the study may be looked at by individuals from Edinburgh Napier University, The Newcastle upon Tyne Hospitals NHS Foundation Trust, Newcastle University and from regulatory authorities. I give permission for these individuals to have access to my data. ☐
4. I understand that notes will be taken during the observations about antibiotic decision-making and that these will be written up, analysed and stored securely and confidentially, and will be destroyed upon completion of the study. ☐
5. I understand that no direct quotations from the observation will be used in reports, academic publications and conference presentations. ☐
6. I agree to take part in the above study. ☐

Thank you for agreeing to take part in this research study.

--	-------------------------	------------------
Name of Person Giving Consent	Signature	Date
--	-------------------------	-------------------
Name of Person Receiving Consent	Signature	Date

FUNDED BY NIHR | National Institute for Health Research

This study is funded by the National Institute for Health Research (NIHR) Health Technology Assessment (HTA) Programme (16/93/01). The views expressed are those of the author(s) and not necessarily those of the NIHR or the Department of Health and Social Care.

Edinburgh Napier UNIVERSITY

Newcastle University

SHORTER TRIAL

A randomised controlled trial of SHORT duration antibiotic thERapy for critically ill patients with sepsis (SHORTER TRIAL)

PROCESS EVALUATION – INFORMATION SHEET (In-Person)

Introduction

You are being invited to contribute to this research study. Before you decide whether or not you would like to take part, it is important for you to understand why the research is being done and what it involves. Please take time to read the following information carefully. Please ask if there is anything that is not clear or if you would like further information. Take as long as you need to decide whether or not you wish to take part.

Study summary

Sepsis is a syndrome of life-threatening organ dysfunction secondary to infection and is a leading cause of death worldwide. A third of patients with sepsis die in hospital and sepsis is estimated to cost the National Health Service (NHS) £15 billion per year. Sepsis is therefore a common reason for admission to a critical care unit, accounting for one-third of general adult critical care admissions, equating to approximately 40,000 patients per year in the UK.

Antibiotics are lifesaving in sepsis and current international and NICE (The National Institute for Health and Care Excellence) guidelines recommend the initiation of antibiotics within one hour of recognition of suspected sepsis. Patients presenting with life-threatening suspected sepsis have broad-spectrum antibiotics initiated in a 'hard-and-fast' approach due to the perceived risk of under-treating a severe infection. Once antibiotics are initiated, the optimal duration is unknown. Previous sepsis guidelines recommended 7-10 days of antibiotics, but updated guidelines encourage shorter duration antibiotics, although this is a weak recommendation based on the very low quality of evidence. UK Health Security Agency (UKHSA) (previously Public Health England) and NICE both promote antibiotic stewardship, encouraging the shortest duration of antibiotics needed and discontinuation of antibiotics if there is no evidence of infection. In this study, we aim to find out if a shorter duration of antibiotics is non-inferior to the usual more than 7-day antibiotic treatment.

We also want to find out nurses' and doctors' views on how easy or difficult it was to follow the protocol. This will give us valuable practical information that can be shared with other units. This part of the study is called a 'process evaluation'. **You are being asked if you are willing to participate in the process evaluation**.

FUNDED BY NIHR | National Institute for Health Research

This study is funded by the National Institute for Health Research (NIHR) Health Technology Assessment (HTA) Programme (16/93/01). The views expressed are those of the author(s) and not necessarily those of the NIHR or the Department of Health and Social Care.

What is the purpose of this study?

This study is intended to help us explore the processes involved in delivering the intervention and any factors and mechanisms of their interaction likely to impact the trial outcomes. Our objectives are to establish the extent to which the intervention is implemented as intended; to ascertain how clinical staff understand and respond to the intervention; to identify barriers and facilitators that affect the implementation of the trial; and explore the importance of the context in the uptake of the intervention.

Why have I been invited?

You have been approached because you are part of the clinical and/or research team directly involved in caring for patients who are enrolled in the study. You have had first-hand experience managing patients with the approaches we are comparing.

Do I have to take part?

No, it is up to you to decide whether you would like to take part. If you agree, you will be given this information sheet to keep and be asked to sign a consent form. If you agree, you are free to withdraw **at any time** without giving a reason.

What will happen if I agree to take part?

We would like to invite you to take part in an individual interview about your experiences and views. These interviews may take place in person within the hospital where you practise, or alternatively, they may take place online via video conferencing facilities or by telephone.

The interview will be guided by a researcher who is experienced in asking healthcare professionals for their views about different ways of delivering care. The aim is to hear your opinions so you will be encouraged to tell us about any issues that you think are relevant to the approaches used in the study. For the purposes of analysing the discussion, we may record what is said using a digital voice recorder and/or take detailed notes. If a recording is taken, this may be typed out word for word so that it can be analysed accurately. Audio-recorded interviews may be transcribed by a professional, external third party transcription service with a duty of confidentiality. Audio-recordings and transcriptions will be stored securely and confidentially. In the instance where direct quotations are used, these will be anonymised.

What are the possible benefits of taking part?

There are no direct benefits to you taking part in this study, but the results from this study might help to improve the healthcare of patients in the future.

What are possible disadvantages of taking part?

There are no known disadvantages to taking part in this study.

What if there are any problems?

If you have a concern about any aspect of this study, please contact: Dr Gosha Colquhoun: G.Colquhoun2@napier.ac.uk or Dr Jamie Smith: J.Smith7@napier.ac.uk who will do their best to answer your questions.

What will happen if I don't want to carry on with the study?

You are free to withdraw your consent to participate in this study **at any time** without giving a reason but we will keep the information we already have.

What happens when the study is finished?

The process evaluation will be conducted during the pilot phase of the study. The interviews will be analysed for themes and the subsequent report will be shared with the trial Chief Investigator, trial management committees and the funder. The results of the research may be published through medical publications. Individual participants will not be identifiable in any published results.

Will taking part in the study be kept confidential?

All the information we collect during the research will be kept confidential and the digital voice recordings will be destroyed upon completion of the study. There are strict laws which safeguard your privacy at every stage. The steps to protect your data are described at the end of this leaflet.

Who is organising and funding the research?

This study is sponsored by the Newcastle upon Tyne Hospitals NHS Foundation Trust. The study is being funded by the National Institute for Healthcare Research – Health Technology Assessment Programme (NIHR HTA).

FUNDED BY NIHR | National Institute for Health Research

This study is funded by the National Institute for Health Research (NIHR) Health Technology Assessment (HTA) Programme (16/93/01). The views expressed are those of the author(s) and not necessarily those of the NIHR or the Department of Health and Social Care.

Who has reviewed the study?

All research in the NHS is looked at by an independent group of people called a Research Ethics Committee. A favourable ethical opinion has been obtained from the Wales REC 4 Research Ethics Committee. NHS Management Approval has also been given. Former participants and relatives and the local Patient Advisory Group also helped us design this trial.

Researcher Contact Details

If you have any further questions about the study please contact Dr Gosha Colquhoun: G.Colquhoun2@napier.ac.uk or Dr Jamie Smith: J.Smith7@napier.ac.uk.

Independent Contact Details

If you would like to discuss this study with someone independent of the study, please contact: Dr Aisling McMahon: AislingMcMahon@mater.ie.

Complaints

If you wish to make a complaint about the study, please contact:

<insert contact details> *to be adapted depending on the research site*.

Further information on Data Protection arrangements

All the information we collect during the research will be kept confidential and there are strict laws to safeguard your privacy at every stage.

All information will be stored by Edinburgh Napier University and Newcastle University. It would only be transferred to other researchers after all necessary approvals were in place.

In order to monitor and audit the study, we will ask your consent for research representatives from the study sponsors to access data collected during the study, where it is relevant to taking part in this research.

How will we use your information?

We will need to use information from you for this research project. This information will include your:

- Name
- Contact details – work telephone number and email address
- Name of institution
- Position in institution

People will use this information to do the research or to make sure that the research is being done properly.

People who do not need to know who you are will not be able to see your name or contact details. Your data will have a code number instead.

Individuals from the Edinburgh Napier University, Newcastle upon Tyne Hospitals NHS Foundation Trust, Newcastle University and regulatory organisations may listen to recordings of interviews/discussions or read the transcripts to check the accuracy of the research study. Audio-recorded interviews may be transcribed by an external third party and will be stored securely and confidentially. The only people in Edinburgh Napier University and Newcastle University, who will have access to information that identifies you, will be people who will audit the data collection process.

We will keep all information about you safe and secure.

Once we have finished the study, we will keep some of the data so we can check the results. We will write our reports in a way that no one can work out that you took part in the study.

FUNDED BY NIHR | National Institute for Health Research

This study is funded by the National Institute for Health Research (NIHR) Health Technology Assessment (HTA) Programme (16/93/01). The views expressed are those of the author(s) and not necessarily those of the NIHR or the Department of Health and Social Care.

Where can you find out more about how your information is used?

You can find out more about how we use your information:

- at www.hra.nhs.uk/information-about-patients/ www.hra.nhs.uk/information-about-patients/
- from our leaflet available from www.hra.nhs.uk/patientdataandresearch
- by asking one of the research team, or
- by contacting the Data Protection Officer at either:
  - Edinburgh Napier University. Email: dataprotection@napier.ac.uk or call 0131 455 2946
  - NHS Lothian Lothian.DPO@nhs.net or call 0131 465 5444

Thank you for your time

FUNDED BY NIHR | National Institute for Health Research

This study is funded by the National Institute for Health Research (NIHR) Health Technology Assessment (HTA) Programme (16/93/01). The views expressed are those of the author(s) and not necessarily those of the NIHR or the Department of Health and Social Care.

Edinburgh Napier UNIVERSITY

Newcastle University

SHORTER TRIAL

A randomised controlled trial of SHORT duration antibiotic thERapy for critically ill patients with sepsis (SHORTER TRIAL)

PROCESS EVALUATION - CONSENT FORM

Centre Name: ____________________

	Please **initial** box
1. I confirm that I have read and understand the Process Evaluation Information Sheet (Version _._, dated _ _ / _ _ / _ _ _ _) for the above study and have had the opportunity to consider the information, ask questions and have these questions answered satisfactorily.	☐
2. I understand that my participation is voluntary and that I am free to withdraw at any time, without having to give a reason for doing so.	☐
3. I understand that relevant sections of my data collected during the study may be looked at by individuals from Edinburgh Napier University, The Newcastle upon Tyne Hospitals NHS Foundation Trust, Newcastle University and from regulatory authorities. I give permission for these individuals to have access to my data.	☐
4. I give permission for my discussion to be recorded, and understand that these recordings may be transcribed by a third party, external to Edinburgh Napier University, The Newcastle upon Tyne Hospitals NHS Foundation Trust and Newcastle University.	☐
5. I understand that the recording will be kept securely and confidentially, and will be destroyed upon completion of the study.	☐
6. I give permission for phrases from my interview to be used in publications and presentations, but understand that my anonymity will be maintained.	☐
7. I agree to take part in the above study.	☐

Thank you for agreeing to take part in this research study.

---	--------------------------	-------------------
Name of Person Giving Consent	Signature	Date
---	--------------------------	--------------------
Name of Person Receiving Consent	Signature	Date

FUNDED BY
NIHR | National Institute for Health Research
This study is funded by the National Institute for Health Research (NIHR) Health Technology Assessment (HTA) Programme (16/93/01). The views expressed are those of the author(s) and not necessarily those of the NIHR or the Department of Health and Social Care.

A randomised controlled trial of SHORT duration antibiotic thERapy for critically ill patients with sepsis (SHORTER TRIAL)

PROCESS EVALUATION – INFORMATION SHEET (Telephone)

This sheet is to help researchers with the process of taking telephone consent from clinical/research staff. Introduce yourself and explain the purpose of the call. The discussion should include the points listed below. Give the staff member the opportunity to ask questions.

Introduction

You are being invited to contribute to this research study. Before you decide whether or not you would like to take part, it is important for you to understand why the research is being done and what it involves. Please take time to read the following information carefully. Please ask if there is anything that is not clear or if you would like further information. Take as long as you need to decide whether or not you wish to take part.

Study summary

Sepsis is a syndrome of life-threatening organ dysfunction secondary to infection and is a leading cause of death worldwide. A third of patients with sepsis die in hospital and sepsis is estimated to cost the National Health Service (NHS) £15 billion per year. Sepsis is therefore a common reason for admission to a critical care unit, accounting for one-third of general adult critical care admissions, equating to approximately 40,000 patients per year in the UK.

Antibiotics are lifesaving in sepsis and current international and NICE (The National Institute for Health and Care Excellence) guidelines recommend the initiation of antibiotics within one hour of recognition of suspected sepsis. Patients presenting with life-threatening suspected sepsis have broad-spectrum antibiotics initiated in a 'hard-and-fast' approach due to the perceived risk of under-treating a severe infection. Once antibiotics are initiated, the optimal duration is unknown. Previous sepsis guidelines recommended 7-10 days of antibiotics, but updated guidelines encourage shorter duration antibiotics, although this is a weak recommendation based on the very low quality of evidence. UK Health Security Agency (UKHSA) (previously Public Health England) and NICE both promote antibiotic stewardship, encouraging the shortest duration of antibiotics needed and discontinuation of antibiotics if there is no evidence of infection. In this study, we aim to find out if a shorter duration of antibiotics is non-inferior to the usual more than 7-day antibiotic treatment.

We also want to find out nurses' and doctors' views on how easy or difficult it was to follow the protocol. This will give us valuable practical information that can be shared with other units. This part

of the study is called a 'process evaluation'. **You are being asked if you are willing to participate in the process evaluation**.

What is the purpose of this study?

This study is intended to help us explore the processes involved in delivering the intervention and any factors and mechanisms of their interaction likely to impact the trial outcomes. Our objectives are to establish the extent to which the intervention is implemented as intended; to ascertain how clinical staff understand and respond to the intervention; to identify barriers and facilitators that affect the implementation of the trial; and explore the importance of the context in the uptake of the intervention.

Why have I been invited?

You have been approached because you are part of the clinical and/or research team directly involved in caring for patients who are enrolled in the study. You have had first-hand experience managing patients with the approaches we are comparing.

Do I have to take part?

No, it is up to you to decide whether you would like to take part. If you agree, you will be given this information sheet to keep and be asked to sign a consent form. If you agree, you are free to withdraw **at any time** without giving a reason.

What will happen if I agree to take part?

We would like to invite you to take part in an individual interview about your experiences and views. These interviews may take place in person within the hospital where you practise, or alternatively, they may take place online via video conferencing facilities or by telephone.

The interview will be guided by a researcher who is experienced in asking healthcare professionals for their views about different ways of delivering care. The aim is to hear your opinions, so you will be encouraged to tell us about any issues that you think are relevant to the approaches used in the study. For the purposes of analysing the discussion, we may record what is said using a digital voice recorder and/or take detailed notes. If a recording is taken, this may be typed out word for word so that it can be analysed accurately. Audio-recorded interviews may be transcribed by a professional external third party transcription service with a duty of confidentiality. Audio-recordings and transcriptions will be stored securely and confidentially. In the instance where direct quotations are used, these will be anonymised.

What are the possible benefits of taking part?

There are no direct benefits to you taking part in this study, but the results from this study might help to improve the healthcare of patients in the future.

What are possible disadvantages of taking part?

There are no known disadvantages to taking part in this study.

What if there are any problems?

If you have a concern about any aspect of this study, please contact: Dr Gosha Colquhoun: G.Colquhoun2@napier.ac.uk or Dr Jamie Smith: Jamie.Smith7@napier.ac.uk who will do their best to answer your questions.

What will happen if I don't want to carry on with the study?

You are free to withdraw your consent to participate in this study **at any time** without giving a reason but we will keep the information we already have.

What happens when the study is finished?

The process evaluation will be conducted during the pilot phase of the study. The interviews will be analysed for themes and the subsequent report will be shared with the trial Chief Investigator, trial management committees and the funder. The results of the research may be published through medical publications. Individual participants will not be identifiable in any published results.

Will taking part in the study be kept confidential?

All the information we collect during the research will be kept confidential and the digital voice recordings will be destroyed upon completion of the study. There are strict laws which safeguard your privacy at every stage. The steps to protect your data are described at the end of this leaflet.

Who is organising and funding the research?

This study is sponsored by the Newcastle upon Tyne Hospitals NHS Foundation Trust.The study is being funded by the National Institute for Healthcare Research – Health Technology Assessment Programme (NIHR HTA).

This study is funded by the National Institute for Health Research (NIHR) Health Technology Assessment (HTA) Programme (16/93/01). The views expressed are those of the author(s) and not necessarily those of the NIHR or the Department of Health and Social Care.

Who has reviewed the study?

All research in the NHS is looked at by an independent group of people called a Research Ethics Committee. A favourable ethical opinion has been obtained from Wales REC 4 Research Ethics Committee. NHS Management Approval has also been given. Former participants and relatives and the local Patient Advisory Group also helped us design this trial.

Researcher Contact Details

If you have any further questions about the study please contact Dr Gosha Colquhoun: G.Colquhoun2@napier.ac.uk or Dr Jamie Smith: J.Smith7@napier.ac.uk.

Independent Contact Details

If you would like to discuss this study with someone independent of the study, please contact: Dr Aisling McMahon: AislingMcMahon@mater.ie.

Complaints

If you wish to make a complaint about the study, please contact:

<insert contact details> *to be adapted depending on the research site*.

FUNDED BY NIHR | National Institute for Health Research
This study is funded by the National Institute for Health Research (NIHR) Health Technology Assessment (HTA) Programme (16/93/01). The views expressed are those of the author(s) and not necessarily those of the NIHR or the Department of Health and Social Care.

Further information on Data Protection arrangements

All the information we collect during the research will be kept confidential and there are strict laws to safeguard your privacy at every stage.

All information will be stored by Edinburgh Napier University and Newcastle University. It would only be transferred to other researchers after all necessary approvals were in place.

In order to monitor and audit the study, we will ask your consent for research representatives from the study sponsors to access data collected during the study, where it is relevant to taking part in this research.

How will we use your information?

We will need to use information from you for this research project. This information will include your:

- Name
- Contact details – work telephone number and email address
- Name of institution
- Position in institution

People will use this information to do the research or to make sure that the research is being done properly.

People who do not need to know who you are will not be able to see your name or contact details. Your data will have a code number instead.

Individuals from the Edinburgh Napier University, Newcastle upon Tyne Hospitals NHS Foundation Trust, Newcastle University and regulatory organisations may listen to recordings of interviews/discussions or read the transcripts to check the accuracy of the research study. Audio-recorded interviews may be transcribed by an external third party and will be stored securely and confidentially. The only people in Edinburgh Napier University and Newcastle University, who will have access to information that identifies you, will be people who will audit the data collection process.

We will keep all information about you safe and secure.

Once we have finished the study, we will keep some of the data so we can check the results. We will write our reports in a way that no one can work out that you took part in the study.

Where can you find out more about how your information is used?

You can find out more about how we use your information:

- at www.hra.nhs.uk/information-about-patients/ www.hra.nhs.uk/information-about-patients/
- from our leaflet available from www.hra.nhs.uk/patientdataandresearch
- by asking one of the research team, or
- by contacting the Data Protection Officer at either:
    - Edinburgh Napier University. Email: dataprotection@napier.ac.uk or call 0131 455 2946
    - NHS Lothian Lothian.DPO@nhs.net or call 0131 465 5444

Thank you for your time

FUNDED BY NIHR | National Institute for Health Research

This study is funded by the National Institute for Health Research (NIHR) Health Technology Assessment (HTA) Programme (16/93/01). The views expressed are those of the author(s) and not necessarily those of the NIHR or the Department of Health and Social Care.

Edinburgh Napier UNIVERSITY

Newcastle University

SHORTER TRIAL

A randomised controlled trial of SHORT duration antibiotic thERapy for critically ill patients with sepsis (SHORTER TRIAL)

PROCESS EVALUATION - CONSENT FORM

Centre Name: ____________________

Script for oral consent (clinical staff)

You have confirmed that you are happy to go through with the interview. What we need to do first is to record verbally your research consent. You have had a chance to read the consent form, and to ask any questions. Are you OK now for me to switch on the recorder to record us going through the consent form?

"Recorder on: this is a consent recording for the SHORTER TRIAL process evaluation study taken on dd/mm/yyyy. Interviewee is: <please state your name>

What I will do now is to read out in turn each statement of the consent form, and then ask is this true for you. All you need to do is answer yes or no to each statement.

1. You have read and understand all of the information provided in the SHORTER Process Evaluation Information Sheet (V_._, dated _ _ / _ _ / _ _ _ _). You have had the opportunity to consider the information and ask questions. If you have asked any questions so far, these questions have been answered satisfactorily.
2. You understand that your participation is voluntary and that you are free to withdraw at any time, without having to give a reason for doing so.
3. You understand that relevant sections of your data collected during the study may be looked at by individuals from Edinburgh Napier University/Newcastle University or from regulatory authorities. You give permission for these individuals to have access to your interview data.
4. You understand that your interview will be audio digitally-recorded and that this recording may be transcribed by a third party external to Edinburgh Napier University/Newcastle University.
5. You understand that the recording will be kept securely and confidentially, and will be destroyed upon completion of the study.
6. You understand and agree to the use of anonymised quotes in publications, reports and other research outputs.
7. You agree to take part in the above study.

Thank you for agreeing to take part in this research study.
