# Supplementary material for: Randomised controlled trial of SHORT duration antibiotic thERapy for critically ill patients with sepsis (SHORTER trial): protocol for a mixed-methods process evaluation
Source: BMJ Open. 2026 Nov 2;16(11):e119173. doi: 10.1136/bmjopen-2026-119173 (PMC13735336; doi:10.1136/bmjopen-2026-119173)
Supplement: Supplementary file 2 [file bmjopen-16-11-s005.pdf]

Online Supplemental File 5

SHORTER Trial Mixed-Methods Process Evaluation (bmjopen-2026-119173)

Contents

1. SHORTER-adapted NoMAD-23 survey instrument (V1.1), adapted from Finch et al. (2018) with intervention-specific wording per the developers' guidance.

2. Baseline site questionnaire (Phase 1, v2.0), developed by the research team informed by CFIR domains and the antimicrobial stewardship literature.

Referenced in Section 4.3.1 of the manuscript.

A RANDOMISED CONTROLLED TRIAL OF SHORT DURATION ANTIBIOTIC THERAPY FOR CRITICALLY ILL PATIENTS WITH SEPSIS (SHORTER TRIAL)

NoMad: Implementation measure based on Normalisation Process Theory[1]

NoMad Survey Instructions

This survey is designed to help get a better understanding of how to apply and integrate new and complex interventions in healthcare.

This survey is part of the SHORTER trial and asks questions about the implementation of **5-day antibiotic prescription in critically ill patients with sepsis**. We understand that people involved with **antibiotic prescription** have different roles, and that people may have more than one role.

From the statements below, please choose an option that best describes ***your main role*** in relation to **antibiotic prescription:**

- **I am involved in managing or overseeing the prescription of antibiotics** ☐
- **I am involved in administering the antibiotics** ☐

For this survey, please answer all the statements from the perspective of this role. Depending on your role or responsibilities in antibiotic practice, some statements may be more relevant than others.

The survey is in 3 parts. Part A asks some brief questions about yourself and your role. Part B includes three general questions about **antibiotic practice in your setting.** Part C contains a set of more detailed questions about **antibiotic adherence OR use.** For each statement in Part C, there is the option to agree or disagree with what is being asked **(OPTION A).** However, if you feel that the statement is not relevant to you, there are also options to tell us why **(OPTION B).**

Please take the time to decide which answer **best suits your experience for each statement and tick the appropriate circle**

[1] Finch, T.L., Girling, M., May, C.R., Mair, F.S., Murray, E., Treweek, S., Steen, I.N., McColl, E.M., Dickinson, C., Rapley, T. (2015). NoMad: Implementation measure based on Normalization Process Theory. [Measurement instrument]. Retrieved from http://www.normalizationprocess.org.

Part A: About yourself

1. What is your gender?

◯ Man ◯ Woman ◯ Transgender ◯ Non-binary ◯ I prefer not to answer

2. How many years have you worked for this critical care unit? *(If your Trust or Health Board has merged with another or changed its name, please include in your answer all the time you have worked with this Trust or Health Board and its predecessors)*

◯ Less than one year ◯ 1-2 years ◯ 3-5 years ◯ 6-10 years ◯ 11-15 years ◯ More than 15 years

3. How would you describe <u>your professional job category</u>?

a. Critical care nurse
b. Advanced Practitioner in critical care
c. Junior doctor in critical care
d. Registrar in critical care
e. Consultant in critical care
f. Pharmacist
g. Physiotherapist
h. Dietician
i. Other (please specify):

Part B: General questions about the intervention

When you use antibiotics, how familiar does it feel?

Still feels very new | **Feels completely familiar**

0 1 2 3 4 5 6 7 8 9 10

Do you feel the decision to prescribe antibiotics is currently a normal part of your work?

Not at all | **Somewhat** | **Completely**

0 1 2 3 4 5 6 7 8 9 10

Do you feel that the decision to prescribe antibiotics will become a normal part of your work?

Not at all | **Somewhat** | **Completely**

0 1 2 3 4 5 6 7 8 9 10

Part C: Detailed questions about the intervention

For each statement, please select an answer that best suits your experience using Option A. If the statement is not relevant to you please select an answer from Option B.

Section C1		Option A					Option B		
		Strongly Agree	Agree	Neither agree nor disagree	Disagree	Strongly disagree	Not relevant to my role	Not relevant at this stage	Not relevant to the intervention
1.	I can see how using antibiotics for ≤ 5 days in critically ill patients with sepsis differs from the usual ways of working.	○	○	○	○	○	○	○	○
2.	Staff in this organisation have a shared understanding of the purpose of prescribing antibiotics for ≤ 5days in critically ill patients with sepsis.	○	○	○	○	○	○	○	○
3.	I understand how prescribing antibiotics for ≤ 5 days in critically ill patients with sepsis affects the nature of my work.	○	○	○	○	○	○	○	○
4.	I can see the potential value of prescribing antibiotics for ≤ 5 days in critically ill patients with sepsis for my work.	○	○	○	○	○	○	○	○

For each statement please select an answer that best suits your experience using Option A. If the statement is not relevant to you, please select an answer from Option B.

Section C2		Option A: Strongly Agree	Agree	Neither agree nor disagree	Disagree	Strongly disagree	Option B: Not relevant to my role	Not relevant at this stage	Not relevant to the intervention
1.	There are key people who drive the decision to prescribe antibiotics for ≤ 5 days in critically ill patients with sepsis forward and get others involved.	○	○	○	○	○	○	○	○
2.	I believe that participating in the decision to prescribe antibiotics for ≤ 5 days in critically ill patients with sepsis is a legitimate part of my role.	○	○	○	○	○	○	○	○
3.	I'm open to working with colleagues in new ways to use antibiotics for ≤ 5 days in critically ill patients with sepsis.	○	○	○	○	○	○	○	○
4.	I will continue to support the decision to prescribe antibiotics for ≤ 5 days in critically ill patients with sepsis.	○	○	○	○	○	○	○	○

For each statement, please select an answer that best suits your experience using Option A. If the statement is not relevant to you, please select an answer from Option B.

Section C3		Option A					Option B		
		Strongly Agree	Agree	Neither agree nor disagree	Disagree	Strongly disagree	Not relevant to my role	Not relevant at this stage	Not relevant to the intervention
1.	I can easily integrate the decision to prescribe antibiotics for ≤ 5 days in critically ill patients with sepsis into my existing work.	○	○	○	○	○	○	○	○
2.	The prescription of antibiotics for ≤ 5 days in critically ill patients with sepsis disrupts working relationships.	○	○	○	○	○	○	○	○
3.	I have confidence in other people's ability to use antibiotics for ≤ 5 days in critically ill patients with sepsis.	○	○	○	○	○	○	○	○
4.	Work is assigned to those with skills appropriate to prescribe antibiotics for ≤ 5 days in critically ill patients with sepsis.	○	○	○	○	○	○	○	○
5.	Sufficient training is provided to enable staff to implement prescription of antibiotics for ≤ 5 days in critically ill patients with sepsis.	○	○	○	○	○	○	○	○
6.	Sufficient resources are available to support the decision to prescribe antibiotics for ≤ 5 days in critically ill patients with sepsis.	○	○	○	○	○	○	○	○
7.	Management adequately supports the decision to	○	○	○	○	○	○	○	○

prescribe antibiotics for ≤ 5 days in critically ill patients with sepsis.	

For each statement please select an answer that best suits your experience using Option A. If the statement is not relevant to you, please select an answer from Option B.

Section C4		Option A: Strongly Agree	Agree	Neither agree nor disagree	Disagree	Strongly disagree	Option B: Not relevant to my role	Not relevant at this stage	Not relevant to the intervention
1.	I am aware of reports about the effects of prescribing antibiotics for ≤ 5 days in critically ill patients with sepsis.	○	○	○	○	○	○	○	○
2.	The staff agree that prescribing antibiotics for ≤ 5 days in critically ill patients with sepsis is worthwhile.	○	○	○	○	○	○	○	○
3.	I value the effects that prescribing antibiotics for ≤ 5 days in critically ill patients with sepsis has had on my work.	○	○	○	○	○	○	○	○
4.	Feedback about prescribing antibiotics for ≤ 5 days in critically ill patients with sepsis can be used to improve antibiotic stewardship in the future.	○	○	○	○	○	○	○	○
5.	I can modify how I work with prescribing antibiotics	○	○	○	○	○	○	○	○

for ≤ 5 days in critically ill patients with sepsis.	

SURVEY CONCLUSION

Thank you for completing our survey.

The SHORTER Trial Research Team

This study is funded by the National Institute for Health Research (NIHR) Health Technology Assessment (HTA) Programme (16/93/01). The views expressed are those of the author(s) and not necessarily those of the NIHR or the Department of Health and Social Care.

A randomised controlled trial of SHORT duration antibiotic thERapy for critically ill patients with sepsis (SHORTER TRIAL)

PROCESS EVALUATION – PHASE I (BASELINE QUESTIONNAIRE)

The SHORTER process evaluation is divided into three phases. Phase I (baseline) is at the beginning of the trial and involves getting a comprehensive picture of organisational structure and usual practice in the participating ICUs. Phases II and III will happen as the trial progresses and will involve speaking to (or observing) staff in a sample of ICUs to understand how the trial is being delivered.

Phase I data are particularly important for the process evaluation as they help us understand the characteristics of participating ICUs. We want to explore the variation in units' resources, usual practices relating to antibiotic prescribing, and any anticipated challenges that staff may perceive. This sets the context for the trial. As the trial progresses, we will examine these organisational and participant-related factors to understand the circumstances under which trial delivery is optimised. Phase I data can help identify the resources necessary to successfully deliver this trial. They are also used to explore how usual practice (what is normally done) impacts on delivering care in a different way (short-duration antibiotic practice).

Thank you for taking the time to complete this questionnaire.

PHASE I QUESTIONNAIRE

Please complete and return this questionnaire to Jamie Smith (Process Evaluation representative) at J.Smith7@napier.ac.uk

1. SITE INFORMATION

Site Name	
Department	
What is your unit speciality?	
How many physical beds are in the unit?<br>⇒ Are all these beds staffed?	
What is the level 2/3 breakdown?	
What is your average **annual** admission rate?	

2. RESEARCH TEAM

**Principal Investigator's Name**		
Contact details	Telephone	
	Email	
**Research Coordinator/Lead Research Nurse for SHORTER**		

FUNDED BY NIHR | National Institute for Health Research

This study is funded by the National Institute for Health Research (NIHR) Health Technology Assessment (HTA) Programme (16/93/01). The views expressed are those of the author(s) and not necessarily those of the NIHR or the Department of Health and Social Care.

Contact details	Telephone	
	Email	
**Research nurses**		
Please list the names of all research nurses who work within your unit.<br><br>For each, describe whether they:<br><br>i) Are full-time or part-time<br>ii) Work solely in the ICU or across multiple units<br>iii) Have previous clinical experience in the ICU		
What total cover for recruitment and research do you currently have?	☐ Monday-Friday daytime working hours<br>☐ 7 days per week daytime working hours<br>☐ 24/7 recruitment<br>☐ Other (please specify):<br><br>______________________________	

3. STAFF NURSES

Please specify **total numbers** of full-time & part-time staff nurses.	
Please describe your unit usage of bank/agency staff nurses.	
What is the average **annual** staff nurse turnover?	

4. DOCTORS

Please specify **total numbers** of junior doctors and consultants on the unit and their roles.	

Can you describe what usual cover is like for:<br><br>i) Day shifts<br>ii) Night shifts<br>iii) Weekends	

5. PHARMACISTS

Is there a single allocated ICU pharmacist on your unit?<br><br>⇒ If yes, please provide the name and contact details.	
What percentage of your pharmacist's role is allocated to ICU?	
Does your pharmacist have an active role in research?<br>⇒ Is there a dedicated clinical trials pharmacist at the site?	
Can you describe what usual cover is like for:<br><br>i) Day shifts<br>ii) Night shifts<br>iii) Weekends	

6. CURRENT ANTIBIOTIC PRACTICE

What is your current standard ICU antibiotic practice?	☐ Prescribe antibiotics empirically on day 1 of a suspected infection<br>☐ Prescribe antibiotics after receiving culture results<br>☐ Prescribe antibiotics only when the patient has a high temperature<br>☐ Other (please specify):<br><br>____________________________________

FUNDED BY NIHR | National Institute for Health Research

This study is funded by the National Institute for Health Research (NIHR) Health Technology Assessment (HTA) Programme (16/93/01). The views expressed are those of the author(s) and not necessarily those of the NIHR or the Department of Health and Social Care.

<table>
<tr><td rowspan="1">Who is involved in prescribing antibiotics for antibiotic treatment?</td><td colspan="3">☐ Medical staff
Pharmacist
Advanced practitioners
Other (please specify):
______________________________</td></tr>
<tr><td rowspan="2">Is there a protocol/policy to guide decision-making in antibiotic prescribing in your ICU?</td><td>Yes</td><td>No</td><td>Comments</td></tr>
<tr><td></td><td></td><td></td></tr>
<tr><td>What is your general unit consensus relating to standard antibiotic duration?</td><td colspan="3">☐ 10 days
☐ 7 days
☐ Individualised approach
☐ Other (please specify):
______________________________</td></tr>
<tr><td>How is antibiotic de-escalation carried out in your ICU?</td><td colspan="3">☐ Individualised approach
☐ Part of a bundle
☐ In accordance with a protocol/policy
☐ Clinical preference
☐ Other (please specify):
______________________________</td></tr>
<tr><td>What is your general unit consensus relating to antibiotic de-escalation?</td><td colspan="3">☐ Continue antibiotics for the full duration
☐ Continue antibiotics unless a negative culture is returned
☐ Other (please specify):
______________________________</td></tr>
</table>

This study is funded by the National Institute for Health Research (NIHR) Health Technology Assessment (HTA) Programme (16/93/01). The views expressed are those of the author(s) and not necessarily those of the NIHR or the Department of Health and Social Care.

7. SEPSIS MONITORING

	Yes	No	Comments
Do your bedside nurses routinely assess for sepsis?			
If yes, what sepsis assessment tool do they use?	☐ NEWS2 score<br>☐ Sepsis 6<br>☐ SOFA score<br>☐ qSOFA score<br>☐ Other (please specify):<br>____		
With what frequency is your sepsis assessment conducted?			

8. SHORTER TRIAL IMPLEMENTATION AND DELIVERY

Regarding facilities or resources at your site, do you anticipate any challenges in implementing the protocol or delivering the intervention?<br><br>⇒ What would they be/why?	
Do you believe there is equipoise on this topic on your unit?<br><br>⇒ Please consider whether you think that clinicians will see the potential value of prescribing antibiotics for ≤ 5 days.	

This study is funded by the National Institute for Health Research (NIHR) Health Technology Assessment (HTA) Programme (16/93/01). The views expressed are those of the author(s) and not necessarily those of the NIHR or the Department of Health and Social Care.

General Comments (If you have anything else to add, which has not been covered by the questionnaire, please do so here)

To the best of my knowledge, the information contained in this questionnaire is correct.

Assessment completed by:

Print Name [Title/Position]	Signature	Date
