# Supplementary material for: Randomised controlled trial of SHORT duration antibiotic thERapy for critically ill patients with sepsis (SHORTER trial): protocol for a mixed-methods process evaluation
Source: BMJ Open. 2026 Nov 2;16(11):e119173. doi: 10.1136/bmjopen-2026-119173 (PMC13735336; doi:10.1136/bmjopen-2026-119173)
Supplement: Supplementary file 3 [file bmjopen-2026-119173.reviewer_comments.pdf]

PEER REVIEW HISTORY

BMJ Open publishes all reviews undertaken for accepted manuscripts. Reviewers are asked to complete a checklist review form and are provided with free text boxes to elaborate on their assessment. These free text comments are reproduced below.

ARTICLE DETAILS

Title (Provisional)

A Randomised Controlled Trial of SHORT Duration Antibiotic thERapy for Critically Ill Patients With Sepsis (SHORTER Trial): Protocol for a Mixed-Methods Process Evaluation

Authors

Smith, Jamie; Mossop, Helen; Walmsley, Zoe; Mawson, Phil; Morton, Miranda; Fernandez-Garcia, Cristina; Emerson, Lydia M; Hancock, Helen C; Teare, Dawn; Ternent, Laura; McMullan, Ronan; Walsh, Timothy S.; Dark, Paul; Simpson, A John; Kumar, Rashmi; Ogden , Margaret; Hellyer, Thomas P; Colquhoun (formerly Wojcik), Dr Gosha

---

VERSION 1 - REVIEW

**Reviewer**	**1**
**Name**	**Scherag, Andre**
**Affiliation**	**Universitatsklinikum Jena**
**Date**	**05-Jun-2026**
**COI**	**None**

This is a study protocol for a process evaluation than should run in parallel to an RCT. Such process evalations are important given that we too frequently learn that implementation of study arms do not result in the expected outcome changes and that it would have been important to know what happend. However, I started reading the protocol expecting that I get information on what is evaluated in the RCT and I was not able to figure out what is done (along the PICO scheme). Then I read that the authors rever to the SPIRIT checklist but again all I found was an adoption to aspects of the process evaluation. By the way there is a 2025 update of SPIRIT. So in a nutshell, if it is not clear what is done in the RCT and what the motivation for the RCT motivation is (beyond just shorter duration of antibiotic therapy (which - by the way - itself makes little sense as it is more important to pick the best antibiotic (with an necessary duration) for a given bug in a given individual)). If this is clear, the process evaluation has a context and can the protocol can also be evaluated better (and will very likely be much less generic than what is presented here). Thank you.

Reviewer **2**

Name **Viswanadham , Ratnalekha**

Affiliation **NYU Grossman School of Medicine, Department of Population Health**

Date **26-Jun-2026**

COI **None**

Overall assessment

The manuscript clearly articulates a relevant implementation question—how a fixed 5-day antibiotic strategy for sepsis is delivered and embedded across ICUs—and proposes a rigorous, theory driven mixed-methods design embedded in an NIHR-funded pragmatic RCT. It adheres to SPIRIT guidance, specifies trial registration and ethics approvals, and explicitly addresses fidelity, separation, and contextual determinants, which is appropriate for a Type 1 effectiveness–implementation hybrid trial. The protocol is methodologically robust, clearly written, and addresses a clinically and policy-relevant question using appropriate implementation science frameworks and a mixed-methods design.

I would recommend minor revisions to clarify operational definitions, further justify certain analytic choices, improve the language, and sharpen the description of how implementation support will be handled during the ongoing trial. The suggested revisions primarily aim to clarify operational definitions (fidelity and implementation distance), make some analytic choices more explicit, and slightly tighten the description of governance and observation procedures. None of these issues undermines the fundamental scientific merit or feasibility of the study.

Major strengths

Importance and relevance

• The question of optimal antibiotic duration for critically ill patients with sepsis is clinically important and highly aligned with antimicrobial stewardship priorities and contemporary sepsis guidelines.

• Embedding a process evaluation that explicitly addresses behavioral, team, and organizational determinants is a strength, particularly given the risk of contamination and evolving "usual care" during an open-label trial.

Conceptual and theoretical framing

• The multilevel use of TPB (micro), NPT (meso), and CFIR (macro) is clearly justified, avoids cross level inference, and is mapped to data sources in both the main text and supplementary materials.

• The logic model (Figure 1) linking context, implementation mechanisms, and outcomes is explicit and consistent with contemporary MRC guidance on process evaluations.

Design and methods

• The longitudinal, convergent mixed methods design with three phases (early delivery, active delivery, sustained delivery) is well described and appropriately aligned to trial timelines.

• The sampling strategy for ~50 sites, including 10 purposively selected case-study ICUs, using maximum variation and "information power," is appropriate and well justified.

• Data collection methods (baseline questionnaires, NoMAD 23 survey, semi structured interviews, structured observations, and routine trial metrics) are described in sufficient detail to permit replication.

• The analysis plan is unusually transparent for a protocol: qualitative framework analysis, construction of composite site scores, operational definitions of fidelity and separation, and planned site level exploratory analyses are all specified a priori.

Governance and ethics

• There is a thorough description of ethics approvals, consent processes (including capacity issues in ICU), data protection, and observational safeguards, which is particularly important for non participant observation in critical care settings.

• The governance protocol (Supplement S4) carefully addresses the boundary between evaluation and facilitation, recognizing the risk that real time process insights could become an additional implementation intervention.

Major points for clarification or improvement

Clarity and feasibility of "fidelity" classification

The operationalization of fidelity (adherent, clinically justified deviation, non adherence) is central to the evaluation but may prove challenging in practice across ~50 sites. It is not fully clear how "documented clinical rationale per the trial's pre specified exception criteria" (after the Table 3 caption) will be operationalized and standardized across sites, especially given heterogeneous documentation practices and varying stewardship maturity. It remains unclear who will adjudicate deviations (e.g., central clinical committee vs. site-level teams) and whether adjudicators will be blinded to outcomes; this matters for consistency and potential information bias.

Suggestion: Add a brief description of (1) the adjudication process for classifying deviations (e.g., centralized review, sample double coding of cases) and (2) any training or calibration for those coding fidelity categories, including how free text rationales in the trial dataset will be interpreted. A table of explicit definitions of fidelity, separation, and variation (your desired metrics in the introduction) would help with replication of your protocol and methods across studies.

Measurement of “implementation distance” and potential collinearity

The composite “implementation distance” score is conceptually useful, but the description suggests some overlap with other composite domains and with outcomes. The score combines current median antibiotic duration, existing short course protocols, and clinician comfort with short courses, all derived from baseline data. There is a risk that this score may be difficult to interpret if it correlates strongly with stewardship infrastructure, practice complexity, or later separation measures; the authors note they will not update it with trial period durations but do not fully address the analytic implications.

Suggestion: Clarify that implementation distance will be used descriptively and for sampling rather than as a predictive covariate in any formal models, and specify how high vs low distance will be operationalized in typology development.

Handling of evolving “usual care” and contamination

The manuscript acknowledges that usual care may drift toward shorter courses and that separation may narrow over time. This is a key risk to interpretability. The plan to use qualitative data to distinguish cross-arm contamination, autonomous drift, and protocol non-delivery is conceptually sound but currently at a high level. It is not entirely clear how time trends in usual care duration will be modeled or visualized, nor how changes in stewardship policies (e.g., the introduction of local short-course guidance) will be captured beyond the initial baseline questionnaire.

Suggestion: Specify more explicitly how temporal changes in usual care duration will be incorporated—for example, whether site level run charts or segmented plots will be generated and how these will be aligned with policy changes identified through documentary analysis. Consider stating that major unit-level changes in antibiotic policy during the trial will be documented (e.g., through periodic document reviews) and incorporated into case summaries.

NoMAD 23 response rates and potential bias

The protocol recognizes that NoMAD response rates are likely to be low and proposes exclusion thresholds (<5 responses or <30% of eligible staff). However, administering NoMAD only once at baseline may miss later changes in coherence or collective action, especially if implementation support or local leadership changes over time. Reliance on baseline perceptions may produce optimistic estimates; while the authors note this, they might further justify why a single baseline measurement is sufficient, given the longitudinal qualitative work.

Suggestion: Briefly justify the choice of a single baseline NoMAD administration in terms of respondent burden and feasibility, and reiterate that longitudinal normalization will primarily be inferred from qualitative data and trial metrics. Consider indicating whether targeted follow-up of particularly low-fidelity sites with abbreviated NPT-based questions was considered, and why it was rejected (if so).

Integration plan and risk of median splits

The integration plan (case summaries, typologies, joint displays) is strong, but the proposed use of median splits to define “high” and “low” fidelity/separation and NoMAD constructs warrants reflection. Median splits can obscure meaningful gradients and reduce interpretability, especially with ~50 sites. Because quantitative indicators will be used primarily to support typology construction rather than inferential modeling, this may be acceptable, but could still lead to arbitrary categorization.

Suggestion: Clarify that median splits will be used as pragmatic thresholds for organizing joint displays rather than for formal hypothesis testing, and that sensitivity analyses using alternative cut points (e.g., quartiles or clinically meaningful thresholds such as ≥ 2-day separation) may be considered. Make sure to find citations to justify these clinically meaningful citations.

Scope and burden of observation

The observation plan (2–3 days per site, including a weekend or handover period) is reasonable but somewhat optimistic about what can be captured, particularly regarding out-of-hours decision-making. The authors note that some dynamics may be missed, but they might further clarify how they will prioritize observation opportunities (e.g., targeting high enrollment periods, times of known antimicrobial review rounds, or specific infection types). Given that observations do not capture direct quotations, there is also a risk that nuance in “prescribing etiquette” or informal sanctioning may be attenuated.

Suggestion: Add one sentence acknowledging that observation field notes, devoid of verbatim quotations, may limit the granularity of analysis of subtle interactional dynamics, and indicate how this will be mitigated (e.g., through immediate post round jotting of paraphrased accounts and triangulation with interviews).

Minor points and editorial suggestions

1. Terminology consistency: Ensure consistent use of terms such as “usual care arm,” “standard care,” “control arm,” and “non intervention arm” across the manuscript to avoid confusion.

2. Clarification of “non participant (i.e., enrolled patient) observation”: The phrase “Non participant (i.e., enrolled patient) observation” in Section 4.3.2 may confuse readers into thinking only trial participants are observed. Consider rephrasing to “non-participant observation of clinical teams caring for enrolled patients.”

3. Gender and SAGER reporting: The description of sex and gender data collection in line with SAGER is commendable; a short statement on how these variables may be used analytically (e.g., to explore whether implementation experiences differ by professional group and gender) would strengthen the justification.

4. Governance protocol redundancy: The governance section and Supplement S4 are lengthy; a brief cross-reference in the main text, noting that details are provided in the supplement, is present but could be more concise to improve readability.

5. Formatting and minor typographical issues: There are occasional small typographical errors (e.g., "Medicla Microbiology" in the author list; inconsistent hyphenation of "short course" vs "short course"). The capitalization and phrasing of objectives and subsections could be more consistent. The SPIRIT checklist in the supplement sometimes references sections by "Section 4.5.1.1," which does not appear explicitly in the main text headings; ensure numbering aligns with the final formatted version.

VERSION 1 - AUTHOR RESPONSE

Reviewer 1 (Prof. Andre Scherag)

R1.1 *"I started reading the protocol expecting that I get information on what is evaluated in the RCT and I was not able to figure out what is done (along the PICO scheme)… if it is not clear what is done in the RCT and what the motivation for the RCT is… the process evaluation has a context and the protocol can also be evaluated better (and will very likely be much less generic than what is presented here)."*

The reviewer is right, and we are grateful for the frankness: this was the most significant weakness in the submitted version. Readers should not need to consult the trial protocol to understand what the host trial does. The Background now includes a summary of the SHORTER trial along PICO lines: adults (≥18 years) in UK critical care units with suspected or confirmed sepsis, randomised within four days of antibiotic initiation (population); a fixed 5-day antibiotic course, with antibiotic choice remaining at the discretion of the clinical team (intervention); usual care, in which duration is determined by the treating team according to best practice guidance, typically around nine days (comparator); and co-primary outcomes of 28-day all-cause mortality (non-inferiority) and total antibiotic treatment days at 28 days (superiority), with a target sample of 2,244 patients across approximately 50 sites (outcomes). We have also expanded the motivation for the trial: short-course evidence from specific infection types largely excluded the sickest ICU patients, so equipoise persists for a fixed-duration strategy in heterogeneous sepsis populations, in a context where prolonged exposure drives avoidable harm and antimicrobial resistance. The summary also now records the trial's NIHR HTA funding (NIHR134101).

On the reviewer's substantive point that choosing the best antibiotic for a given organism and individual matters more than duration: we agree that appropriate selection is essential, and the trial does not constrain it. Choice, escalation and de-escalation remain with the clinical team in both arms. The trial intentionally isolates antibiotic duration rather than antibiotic selection because antibiotic choice is already governed by antimicrobial stewardship processes, whereas optimal duration remains uncertain and highly variable; we see the two questions as complementary rather than competing. This clarification has been added to Section 1. We hope the process evaluation now reads as considerably less generic with the host trial properly in view.

R1.2 *"…the authors refer to the SPIRIT checklist but again all I found was an adaption to aspects of the process evaluation. By the way there is a 2025 update of SPIRIT."*

Thank you for pointing us to the update. The protocol now follows SPIRIT 2025 (Chan et al., BMJ 2025;389:e081477). The checklist has been remapped to the 34-item SPIRIT 2025 structure,

including the new open science and patient and public involvement items, with the process-evaluation adaptations annotated: items concerning randomisation, blinding and clinical harms pertain to the host trial and are cross-referenced to the trial protocol rather than duplicated. The revised checklist replaces the previous supplemental file, and its section references now align with the manuscript's headings.

Reviewer 2 (Dr Ratnalekha Viswanadham)

We thank the reviewer for a careful and constructive review. Nearly all of the suggestions have been adopted, and the protocol is better for them.

R2.1 *Clarity and feasibility of "fidelity" classification: adjudication, training and calibration, interpretation of free-text rationales; a table of explicit definitions of fidelity, separation and variation.*

Section 4.4.2 now describes the adjudication process: fidelity categories will be assigned centrally by the process evaluation team using the trial's pre-specified exception criteria and a written coding manual, blind to site identity and clinical outcomes; free-text rationales will be coded against the manual; a random 10% sample will be independently double-coded (JBS, GC), with disagreements resolved by discussion and consensus, and referred to the Chief Investigator where clinical judgement is required; and calibration exercises will precede each analysis phase. As suggested, a table of operational definitions of fidelity, separation and variation has been added as Table 4 to support replication. This was a helpful suggestion, and we expect the table to be of practical use during analysis.

R2.2 *Measurement of "implementation distance" and potential collinearity.*

Clarified as suggested (Section 4.4.2). Implementation distance will be used descriptively and to inform purposive sampling only, and will not enter any model as a predictive covariate. For typology development, high and low implementation distance will be defined by median split across sites, consistent with the treatment of the other composite indicators. In making this clarification we also reviewed the scoring materials against the data source: the composite-score components in online supplemental Table S5, Table 4 and Supplement S1 (Table S3) have been aligned with the items actually collected in the baseline site questionnaire (Supplement S5), and free-text questionnaire items (anticipated challenges; perceived equipoise) are now explicitly coded qualitatively rather than scored.

R2.3 *Handling of evolving "usual care" and contamination: temporal modelling and visualisation, and capture of policy changes beyond baseline.*

We agree this needed more specificity. Section 4.4.2 now states that temporal change in usual-care antibiotic duration will be visualised using site-level run charts of monthly median duration with trial-phase segmentation, annotated with the timing of relevant local policy changes; and that unit-level changes in antibiotic policy during the trial will be captured through document review at each case-study visit and a brief annual document request to all sites, feeding into site case summaries and joint displays.

R2.4 *NoMAD-23 response rates and potential bias: justify the single baseline administration; state whether targeted follow-up of low-fidelity sites was considered.*

Section 4.4.2 now gives the justification. A single baseline administration will provide an exploratory, descriptive assessment of staff expectations about how implementation may work while limiting respondent burden on a workforce with high survey exposure and constrained capacity; repeated administration risks progressively unrepresentative responses as engaged staff self-select. The SHORTER-adapted wording has not been separately validated, and the survey is not used as a longitudinal measure; change in implementation over time will instead be examined through interviews, observations and trial metrics. We did consider targeted follow-up of low-fidelity sites

with abbreviated NPT-based questions and rejected it, because differential measurement across sites would compromise comparability. Phase 3 interviews already prioritise sites with contrasting trajectories and provide the same insight qualitatively. This calibrated role is now stated in the manuscript.

R2.5 *Integration plan and risk of median splits.*

We agree this warranted clarification. Section 4.4.3 now clarifies that median splits serve as pragmatic thresholds for organising joint displays and typology construction, not for hypothesis testing, and that sensitivity analyses will examine alternative cut points, including quartiles and a pragmatic sensitivity threshold of at least 2 days, selected relative to the anticipated contrast between the fixed 5-day course and usual-care durations of approximately 9 days assumed in the host trial design. This clarification should make the analytical purpose of these thresholds clearer and avoid the impression that they are intended for inferential analyses.

R2.6 *Scope and burden of observation: acknowledge limits of non-verbatim field notes and describe mitigation.*

Added almost verbatim, as the reviewer put it well (Section 4.3.3): because field notes will not record verbatim speech, subtle interactional dynamics such as “prescribing etiquette” (implicit norms governing prescribing decisions) and informal sanctioning may be attenuated; we will mitigate this through immediate post-observation recording of paraphrased accounts and triangulation with interview data. We have also noted that observation windows will prioritise antimicrobial review rounds and periods of active recruitment.

Minor points

R2.m1 Terminology has been standardised to "usual care" and "usual care arm" throughout. The single instance of "standard care" in the Background has been changed, and no instances of "control arm" or "non-intervention arm" remained.

R2.m2 "Non-participant (i.e., enrolled patient) observation" has been rephrased to "non-participant observation of clinical teams caring for enrolled patients", exactly as suggested (Section 4.3.3).

R2.m3 On SAGER: Section 4.2.2 now states that analysis will consider whether implementation experiences differ by professional group, seniority and gender.

R2.m4 The governance paragraph in Section 4.1 has been condensed, retaining the essential safeguards and cross-referencing Supplement S4 and Table 2 for the detail.

R2.m5 Thank you for spotting these. "Medicla Microbiology" and the related misspellings sit in the submission system's author metadata rather than the manuscript file and will be corrected in the ScholarOne author records as part of this resubmission. Hyphenation of "short-course" as a modifier has been standardised, two garbled sentences repaired, and capitalisation of headings made consistent. The SPIRIT checklist section references now align with the manuscript's heading numbering; the "Section 4.5.1.1" style references were an artefact of an earlier draft, and the reviewer was right to flag them.

Additional changes made in finalising the resubmission

Before the prior resubmission, we audited the generated submission proof and made the following further corrections. The changes listed below remain visible in the marked copy.

1. The published SHORTER host-trial protocol is now cited alongside the trial registration (Mawson et al., 2026), at the host-trial summary, the intervention specification and the Trial Status section, and a reference for the SAGER guidelines has been added (Heidari et al., 2016).

2. Ethics identifiers have been standardised throughout: favourable ethical opinions were received from Wales REC 4 (REC reference 23/WA/0197) and Scotland A Research Ethics Committee (REC reference 24/SS/0013), and Health Research Authority approval is recorded under IRAS ID 317788.

3. The Patient and Public Involvement statement has been moved into the Methods section (new Section 4.7), in line with journal policy, and expanded against the journal's standard PPI questions.

4. A guarantor, a statement on the roles of the funder and sponsor, and an AI-use declaration have been added to the statements. The declaration records that Claude (Anthropic) and Codex (OpenAI) assisted with drafting and revising text, document formatting, consistency and reference checking, and preparation of submission materials; it also confirms that no AI tool generated or analysed study data, that all AI-assisted content was critically reviewed against source materials, and that the authors take full responsibility for the manuscript.

5. The timeline description has been clarified: data collection spans Months 1–30 (December 2024 to May 2027); integration and synthesis follow recruitment closure (30 June 2027) and quantitative dataset lock; reporting aligns to trial completion (May 2028). Figure 2 has been redrawn accordingly. Figure 1 has been redrawn at print resolution and restructured so that its columns follow the manuscript directly: intervention and site context, the three-level theoretical mechanisms, the operationalised delivery outcomes of Table 4 (fidelity, separation, usual-care dynamics, trial delivery, variation), and the host-trial endpoints, with data sources and the integration sequence shown beneath; its conceptual content is retained and the trial logo has been removed in line with figure conventions.

6. The site-profile scoring rubric (Supplement S1, Table S3) now specifies item-level codes, normalisation, tertile derivation and missing-item rules; the NoMAD site-inclusion rules in Section 4.4.2 and Supplement S1 have been consolidated into a single pre-specified hierarchy, including handling of the instrument's 'not relevant' response options; missing-data rules for routine trial data have been added; and the exploratory analyses in Supplement S1 are limited to the two associations pre-specified in the main text.

7. Section 4.3.1 now notes that the adapted NoMAD items use the wording presented to respondents ("prescribing antibiotics for five days or fewer"), whereas the trial defines adherence as exactly five 24-hour periods from the first antibiotic dose and the stop date/time may fall on trial day 6. The section also notes that three general items retain the instrument's generic wording and that the psychometric properties of the adaptation have not been separately established; the instrument is supplied as administered in Supplement S5.

8. Minor corrections: a comma splice repaired; the interview-incentive statement moved to the present tense; observation visits stated as 2–3 days within the REC-approved maximum of five; the anticipated participant number clarified as unique participants; the interquartile-range comparison specified as the difference between arms; the published spelling restored in the Chastre et al. reference and an incorrect chapter DOI removed from the Ritchie and Spencer reference; the Wrocław Medical University affiliation completed; and the SPIRIT 2025 checklist updated to match, including marking biological-specimen provisions not applicable. During final pre-submission checks, reference titles and product names were aligned with authoritative records (including Normalization Process Theory and MAXQDA 24), the Data Protection Act entry was corrected, and journal references were normalised to BMJ house style.

9. Supplement S2 now reproduces the current approved participant information sheets and consent forms exactly as approved (observation PISCF version 2.0; in-person and telephone interview PISCFs version 3.0; all dated 24 September 2025), replacing an earlier working copy in which hyperlinked contact details had been lost; site-adaptable fields appear as in the approved masters. Supplement S2 is supplied as a PDF to preserve the approved documents' formatting. The data-retention statement in Section 5 has been aligned with the approved consent commitments: audio recordings

and observation field notes are destroyed on completion of the study, while pseudonymised interview transcripts are retained in accordance with the sponsor-approved retention schedule.

10. The main-text word count is now 4,314 (sections 1–6, including headings and narrative notes, and excluding the abstract, table cells and captions, figure placeholders, references and end matter; whitespace-delimited count). We note the current Instructions for Authors do not specify a numeric limit for study protocols; we are happy to trim if the editors prefer.

11. Final source-verification refinements have been made: the published host-trial manuscript is now identified as based on protocol version 6.0 (24 September 2025); the intervention and fidelity definitions now use the exact five × 24-hour course and classify any longer or shorter course as non-compliance while coding documented reasons separately; the baseline NoMAD-23 analysis is characterised conservatively as an exploratory, descriptive assessment of staff expectations rather than a validated readiness or anticipated-normalisation measure; and the Background citation attribution now separates clinical-duration factors from microbiology advice, team hierarchy and multidisciplinary influence.
