# Supplementary material for: Randomised controlled trial of SHORT duration antibiotic thERapy for critically ill patients with sepsis (SHORTER trial): protocol for a mixed-methods process evaluation
Source: BMJ Open. 2026 Nov 2;16(11):e119173. doi: 10.1136/bmjopen-2026-119173 (PMC13735336; doi:10.1136/bmjopen-2026-119173)
Supplement: Supplementary file 4 [file bmjopen-16-11-s001.docx]

# Supplement S1: Detailed Analysis Plan for the SHORTER Trial Process Evaluation

## S1.1 Overview and Purpose

This supplement provides additional detail on the planned qualitative, quantitative and mixed-methods integration approaches for the embedded process evaluation within the SHORTER trial (ISRCTN40090372). The process evaluation focuses on intervention delivery (including fidelity and separation), trial delivery, and mechanisms/context influencing delivery across UK intensive care units. The analysis plan was developed prior to analysis and is intended to support transparency and reproducibility.

## S1.2 Theoretical Framework and Operationalisation

The process evaluation is guided by a multi-level framework spanning micro (individual), meso (team/work) and macro (organisational/system) levels. Table S1 summarises the operationalisation of key constructs and the data sources used to examine them.

### Table S1. Theoretical framework, operationalisation and data sources

| Level | Framework | Key Constructs | Operationalisation | Data Sources |
| --- | --- | --- | --- | --- |
| Micro (Individual) | Theory of Planned Behaviour (TPB) | Attitudes | Beliefs about outcomes of stopping antibiotics at day 5 | Interviews |
|  |  | Subjective norms | Perceived social pressure from colleagues, guidelines, patients/families | Interviews |
|  |  | Perceived behavioural control | Confidence in ability to stop antibiotics; perceived barriers | Interviews |
|  |  | Intention | Stated intention to follow the 5-day protocol | Interviews |
| Meso (Team) | Normalisation Process Theory (NPT) | Coherence | Understanding of the intervention and its purpose | NoMAD-23; Interviews; Observations |
|  |  | Cognitive participation | Engagement and commitment to the intervention | NoMAD-23; Interviews; Observations |
|  |  | Collective action | Work done to enact the intervention | NoMAD-23; Interviews; Observations |
|  |  | Reflexive monitoring | Appraisal of the intervention’s effects | NoMAD-23; Interviews; Observations |
| Macro (Organisation) | Consolidated Framework for Implementation Research (CFIR) | Inner setting | Structural characteristics, networks, culture, implementation climate | Site questionnaire; Documents; Interviews |
|  |  | Outer setting | Patient needs, external policies, peer pressure | Documents; Interviews |
|  |  | Intervention characteristics | Adaptability, complexity, relative advantage | Interviews |
|  |  | Characteristics of individuals | Knowledge, self-efficacy | Interviews |
|  |  | Process | Planning, engaging, executing, reflecting | Interviews; Trial metrics  Note: observations primarily evidence collective action (the enacted work of delivery); observational data bearing on other NPT constructs are cross-coded under the decision rule in Section 4.4.1 of the main text. |

## S1.3 Qualitative Analysis

Interview transcripts, observation field notes and relevant documents (e.g., local antimicrobial stewardship policies, guidelines and training materials) will be analysed using Framework Analysis. This approach supports structured cross-case comparison while allowing both deductive analysis using TPB/NPT/CFIR constructs and inductive coding for themes.

### Table S2. Qualitative Analysis Plan

| Stage | Activity | Output |
| --- | --- | --- |
| 1. Familiarisation | Reading and re-reading transcripts, field notes, documents; noting initial impressions | Familiarisation notes |
| 2. Coding framework development | Developing initial framework based on TPB/NPT/CFIR; remaining open to inductive codes | Draft coding framework |
| 3. Indexing | Systematic application of codes to all data using MAXQDA 24 | Coded dataset |
| 4. Charting | Creating framework matrices organised by site and theme | Framework matrices |
| 5. Mapping and interpretation | Identifying patterns, explanations, typologies | Thematic findings; cross-site comparisons |

### Quality Assurance Procedures

- Analyst training and calibration sessions before coding begins
- Double-coding of ~20% of transcripts/field notes by two researchers
- Iterative refinement of coding framework through regular analysis meetings
- Audit trail documenting coding decisions and framework changes
- Reflexive debriefing after each site visit and analysis phase
- Member checking with purposive sub-sample of participants

## S1.4 Quantitative Analysis

### S1.4.1 Baseline site profiling (composite scores)

Baseline data from the pre-trial site questionnaire will be used to generate four composite site scores (0–10 each): antimicrobial stewardship infrastructure, research infrastructure, unit complexity, and implementation distance. These scores will support (i) purposive sampling for the qualitative case-study sites and (ii) contextual description for cross-site comparison.

### Table S3. Site profile scoring rubric

| Domain | Component Items | Scoring Approach |
| --- | --- | --- |
| **Antimicrobial Stewardship Infrastructure** (0-10) | Dedicated ICU pharmacist (Y/N); pharmacist time allocated to ICU (%); antibiotic prescribing protocol/policy in place (Y/N); structured de-escalation approach (protocol/bundle vs individualised) | Items: dedicated ICU pharmacist (Y=1/N=0); pharmacist ICU time (site tertiles: 0/0.5/1); prescribing protocol/policy in place (Y=1/N=0); de-escalation approach (protocol/policy or bundle=1; individualised or clinical preference=0). Domain score = mean of completed item scores × 10. |
| **Research Infrastructure** (0-10) | Research nurse staffing (number, full/part-time, ICU experience); extent of recruitment cover (weekday daytime / 7-day daytime / 24-7); dedicated clinical trials pharmacist (Y/N); pharmacist active role in research (Y/N) | Items: research nurse provision, averaged over three sub-items (staffing numbers in site tertiles 0/0.5/1; any full-time research nurse Y=1/N=0; any ICU-experienced research nurse Y=1/N=0); recruitment cover (Mon–Fri daytime=0; 7-day daytime=0.5; 24/7=1); dedicated clinical trials pharmacist (Y=1/N=0); pharmacist active in research (Y=1/N=0). Domain score = mean of completed item scores × 10. |
| **Unit Complexity** (0-10) | Staffed bed numbers (tertiles); level 2/3 case mix; annual admission rate; unit speciality | Items: staffed beds (site tertiles: 0/0.5/1); level 3 proportion of level 2/3 case mix (site tertiles); annual admissions (site tertiles); unit speciality (general mixed=0; specialist=1). Domain score = mean of completed item scores × 10. |
| **Implementation Distance** (0-10) | Unit consensus on standard antibiotic duration (10 days / 7 days / individualised); antibiotic prescribing/de-escalation protocol in place (Y/N); unit consensus on de-escalation (continue full course vs stop on negative cultures) | Items: unit consensus on standard duration (7 days=0; individualised=0.5; 10 days=1); prescribing/de-escalation protocol in place (No=1/Yes=0, absence indicating no structured mechanism through which the 5-day rule can be embedded); de-escalation consensus (stop on negative cultures=0; continue full course=1). Domain score = mean of completed item scores × 10; higher = greater change required to reach the 5-day target.  Scoring rules: binary items are coded 1/0 and ordinal items are assigned equally spaced codes between 0 and 1 as listed; tertile boundaries are computed across all participating sites' baseline returns and reported with results. 'Other (please specify)' responses are assigned to the nearest listed category by two researchers, with disagreements resolved by discussion; free-text items are coded qualitatively and are not scored. A domain score is computed when at least 75% of its items are complete, as the mean of completed item scores rescaled to 0–10; otherwise the domain is recorded as missing and the site is flagged in reporting. |

### S1.4.2 NoMAD-23 analysis (baseline)

The NoMAD-23 survey will be administered once at baseline across participating sites for an exploratory, descriptive assessment of staff expectations about how implementation may work across NPT constructs. Analysis will include:

- **Individual-level:** Construct means (1–5) with respondent counts
- **Site-level: Construct means calculated for sites meeting the pre-specified inclusion hierarchy below**
- **Visualisation:** Radar plots showing baseline staff-expectation profiles by site
- **Exploratory associations: two pre-specified site-level associations, namely stewardship infrastructure score with site-level fidelity, and NoMAD collective action score with site-level separation (main text, Section 4.4.2); any further associations will be explicitly labelled post hoc**

Inclusion hierarchy (pre-specified): (i) eligible respondents are clinical and research staff involved in antibiotic decision-making or trial delivery at the site; (ii) a returned survey counts as a valid response when at least 75% of the 20 NPT construct items carry a substantive (Option A) answer, and Option B answers ('not relevant to my role', 'not relevant at this stage', 'not relevant to the intervention') are recorded as not applicable and excluded from construct means, which are computed over non-missing items; (iii) site-level construct means are reported where a site has at least five valid responses and a response rate of at least 30% of eligible staff; (iv) sites failing either threshold are excluded from NoMAD-based cross-site comparisons and characterised qualitatively (main text, Section 4.4.2). The three general (Part B) items are reported descriptively and do not contribute to construct means.

### S1.4.3 Implementation and trial-delivery metrics

Routine trial data will be used to summarise, by site and over time:

- Recruitment rate (randomised/eligible)
- Data completeness
- Protocol deviations (counts and types)
- Antibiotic duration (intervention and usual care arms)
- Fidelity (ITT and evaluable denominators)
- Separation (median difference between arms)

Missing-data handling: completeness of routine trial data will be reported monthly by site; patients whose antibiotic-duration data are insufficient to classify fidelity will be reported as unclassifiable and retained in intention-to-treat denominators, with evaluable-denominator analyses excluding them and counts reported. Site questionnaire missing-data rules follow Table S3.

## S1.5 Mixed-Methods Integration and Synthesis

Integration will occur through a staged convergent mixed-methods synthesis:

1. **Within-site case summaries** combining baseline context (documents and composite scores), staff expectations (NoMAD), qualitative findings (TPB/NPT/CFIR) and monthly delivery metrics
2. **Cross-site comparison** to identify implementation trajectory typologies (e.g., high baseline staff expectations/high fidelity; improving over time; persistently low separation)
3. **Joint displays** aligning quantitative indicators with qualitative explanations by construct and time phase
4. **Meta-inference generation** examining convergence, complementarity and discordance

## S1.6 Transparency, Quality Assurance and Deviations

We will maintain:

- Version-controlled analysis log documenting coding framework changes, aggregation decisions, and deviations from this plan (with dates and rationale)
- Archived quantitative code for reproducibility
- Reflexive notes and team debrief summaries
- Documentation of any protocol amendments
