# Supplementary material for: Randomised controlled trial of SHORT duration antibiotic thERapy for critically ill patients with sepsis (SHORTER trial): protocol for a mixed-methods process evaluation
Source: BMJ Open. 2026 Nov 2;16(11):e119173. doi: 10.1136/bmjopen-2026-119173 (PMC13735336; doi:10.1136/bmjopen-2026-119173)
Supplement: Supplementary file 5 [file bmjopen-16-11-s003.docx]

# Supplement S3: Structured Observation Tool

*SHORTER Trial Process Evaluation — Observational Data Collection Tool (V1.0)*

This structured observation tool guides non-participant observation of ward rounds, multidisciplinary meetings and handovers in case-study ICU sites. It focuses on decision-making processes and implementation work rather than quality-of-care audit. Two sections are provided: (1) Routine Clinical Practice, capturing baseline prescribing behaviour and team dynamics; and (2) Intervention/Trial Delivery, capturing organisational and participant-related factors influencing SHORTER trial implementation.

## Section 1: Routine Clinical Practice

| **SESSION DETAILS** | **SESSION DETAILS** | **SESSION DETAILS** | **SESSION DETAILS** | **SESSION DETAILS** |
| --- | --- | --- | --- | --- |
| **Date:** | _____ / _____ / _____ | _____ / _____ / _____ | **Start time:** | _____ : _____ |
| **Observer:** | *Initials*: | *Initials*: | **Finish time:** | _____ : _____ |
| **Episode:** | _____ of _____ | _____ of _____ | **Setting:** |  |
| **Interaction observation type** | Medical  Nursing  MDT  Other (please specify):  _________________________________________ | Medical  Nursing  MDT  Other (please specify):  _________________________________________ | Medical  Nursing  MDT  Other (please specify):  _________________________________________ | Medical  Nursing  MDT  Other (please specify):  _________________________________________ |
| **Staff observed** | Consultant  Junior doctor  Nurse in charge  Bedside nurse Band: ______  Pharmacist  Other HCP (please specify):  _________________________________________ | Consultant  Junior doctor  Nurse in charge  Bedside nurse Band: ______  Pharmacist  Other HCP (please specify):  _________________________________________ | Consultant  Junior doctor  Nurse in charge  Bedside nurse Band: ______  Pharmacist  Other HCP (please specify):  _________________________________________ | Consultant  Junior doctor  Nurse in charge  Bedside nurse Band: ______  Pharmacist  Other HCP (please specify):  _________________________________________ |
| **Development of care plan** | Who is involved in the decision-making process?  *Consider hierarchy.* |  |  |  |
| **Development of care plan** | Is the decision guided by embedded practice or policy/guideline driven?  *Consider:*  *Prescribing etiquette*  *Are decisions based on the unit consensus approach?* |  |  |  |
| **Development of care plan** | Are plans for de-escalation/review discussed? |  |  |  |
| **Communication**  **style** | Type | Question  Discussion  Order | Question  Discussion  Order | Question  Discussion  Order |
| **Communication**  **style** | Tone | Friendly  Strained  Professional  Dismissive  Other (please specify):  ______________________________ | Friendly  Strained  Professional  Dismissive  Other (please specify):  ______________________________ | Friendly  Strained  Professional  Dismissive  Other (please specify):  ______________________________ |
| **Ward round discussions** | Is antibiotic continuation / de-escalation reviewed? How? |  |  |  |
| **Ward round discussions** | What factors are considered in the (dis)continuation of antibiotics? |  |  |  |
| **Ward round discussions** | Who is involved in the decision-making process? |  |  |  |
| **Communication**  **style** | Type | Question  Discussion  Order | Question  Discussion  Order | Question  Discussion  Order |
| **Communication**  **style** | Tone | Friendly  Strained  Professional  Dismissive  Other (please specify):  ______________________________ | Friendly  Strained  Professional  Dismissive  Other (please specify):  ______________________________ | Friendly  Strained  Professional  Dismissive  Other (please specify):  ______________________________ |
| **Other considerations:** | *Patient / unit acuity*  *Skill-mix*  *Resources* |  |  |  |

## Section 2: Intervention / Trial Delivery

| **SESSION DETAILS** | **SESSION DETAILS** | **SESSION DETAILS** | **SESSION DETAILS** | **SESSION DETAILS** |
| --- | --- | --- | --- | --- |
| **Date:** | _____ / _____ / _____ | _____ / _____ / _____ | **Start time:** | _____ : _____ |
| **Observer:** | *Initials*: | *Initials*: | **Finish time:** | _____ : _____ |
| **Episode:** | _____ of _____ | _____ of _____ | **Setting:** |  |
| **Interaction observation type** | Medical  Nursing  MDT  Other (please specify):  _________________________________________ | Medical  Nursing  MDT  Other (please specify):  _________________________________________ | Medical  Nursing  MDT  Other (please specify):  _________________________________________ | Medical  Nursing  MDT  Other (please specify):  _________________________________________ |
| **Staff observed** | Consultant  Junior doctor  Nurse in charge  Bedside nurse Band: ______  Pharmacist  Other HCP (please specify):  _________________________________________ | Consultant  Junior doctor  Nurse in charge  Bedside nurse Band: ______  Pharmacist  Other HCP (please specify):  _________________________________________ | Consultant  Junior doctor  Nurse in charge  Bedside nurse Band: ______  Pharmacist  Other HCP (please specify):  _________________________________________ | Consultant  Junior doctor  Nurse in charge  Bedside nurse Band: ______  Pharmacist  Other HCP (please specify):  _________________________________________ |
| **Organisational factors**  *(how / why is delivery (not) achieved?)* | Unit culture  *Facilitation strategies:*  *Are staff aware?*  *Is there evidence of active education / support?* |  |  |  |
| **Organisational factors**  *(how / why is delivery (not) achieved?)* | Organisational structure  *How does the unit appear is terms clinical and research team provision?*  *Is the unit specialty or patient cohort having an impact?* |  |  |  |
| **Organisational factors**  *(how / why is delivery (not) achieved?)* | Resources  *Does the unit appear to be under-resourced?*  *Consider clinical / research team.* |  |  |  |
| **Participant-related factors**  *(how / why is delivery (not) achieved?)* | Attitudes & perceptions  *Who is involved?*  *Is there discussion / evidence of any of the following:*  *Risk / benefit*  *Impact of usual practice*  *Patient suitability*  *Clinician prescribing preferences*  *Willingness / resistance to change*  *Patient / relative influence* |  |  |  |
| **Comments / impressions** |  |  |  |  |

*Note: This tool is designed to structure field note collection during non-participant observations. Researchers record handwritten notes during observations and complete structured sections as soon as practicable afterwards. All data are pseudonymised; no patient-identifiable information is recorded. Observation safeguards and escalation procedures are detailed in Supplement S2.*
