# Supplementary material for: Randomised controlled trial of SHORT duration antibiotic thERapy for critically ill patients with sepsis (SHORTER trial): protocol for a mixed-methods process evaluation
Source: BMJ Open. 2026 Nov 2;16(11):e119173. doi: 10.1136/bmjopen-2026-119173 (PMC13735336; doi:10.1136/bmjopen-2026-119173)
Supplement: Supplementary file 6 [file bmjopen-16-11-s004.docx]

# Supplement S4: Process Evaluation Governance Protocol

## S4.1 Purpose and Scope

This supplement details the governance arrangements for managing the boundary between evaluation and facilitation within the SHORTER trial process evaluation. Because the process evaluation runs concurrently with an open-label pragmatic trial, the research team will generate real-time knowledge about implementation processes, site-level challenges, and variation in delivery. Without explicit governance, this knowledge could be used — intentionally or inadvertently — to facilitate implementation in ways that constitute an additional intervention, compromising the trial's internal validity and the interpretability of process evaluation findings. The protocol below specifies what information may be shared, with whom, in what form, and under what conditions.

## S4.2 Principles

Three principles govern information flow between the process evaluation and the trial:

1. **Separation of evaluation and facilitation.** The process evaluation team will not provide site-specific implementation support, coaching, or tailored feedback designed to improve delivery at individual sites. Implementation support will be delivered through standard trial monitoring processes (Trial Manager, site PI communications, routine trial teleconferences) and documented in the trial's support log.
2. **Aggregate, descriptive feedback only.** Feedback to the Trial Management Group (TMG) will be limited to aggregate quantitative indicators and descriptive summaries across sites. Individual site performance will not be singled out in TMG reports, and qualitative data (interview excerpts, observation field notes) will not be shared with the TMG during the trial.
3. **Proportionate exception for safety and protocol integrity.** Where the process evaluation identifies a serious protocol violation, patient safety concern, or systematic deviation that could compromise trial integrity across multiple sites, the Chief Investigator and Trial Steering Committee (TSC) will be informed. This exception pathway is pre-specified below (S4.5).

## S4.3 Permitted Information Sharing

The following information may be shared by the process evaluation team with the TMG during the trial recruitment period:

- Aggregate fidelity and separation metrics across all sites (monthly, derived from routine trial data).
- Summary descriptive statistics from the baseline site questionnaire (composite scores, NoMAD-23 construct means across sites).
- General thematic observations about implementation at the programme level (e.g., "several sites report uncertainty about stopping criteria for patients with specific infection types"), presented without site identification and without interpretive claims about causes or solutions.
- Recruitment and data completeness indicators (already available to the TMG through routine trial monitoring).

## S4.4 Restricted Information

The following information will not be shared with the TMG or site teams during the trial:

- Site-specific qualitative findings (interview themes, observation notes, or case summaries identifiable to individual sites).
- Interpretive or explanatory analyses (e.g., proposed mechanisms linking context to fidelity, typologies of implementation trajectories).
- Comparative site performance rankings or league tables.
- Recommendations for site-specific implementation strategies, tailored training, or targeted facilitation.

These data will be analysed and reported after the trial's primary endpoint data are locked, or earlier only through the exception pathway (S4.5).

## S4.5 Exception Pathway

If the process evaluation team identifies any of the following during data collection, the pre-specified escalation pathway will be activated:

- A serious and systematic protocol violation at one or more sites that could affect patient safety or data integrity.
- Evidence of a systematic misunderstanding of the intervention protocol that, if uncorrected, would compromise the trial's ability to test its primary hypothesis.
- A patient safety concern observed during site visits (handled per the observation escalation pathway in Supplement S2, §S2.5).

Escalation will follow this sequence: (i) the process evaluation lead (GC) will notify the Chief Investigator (TPH); (ii) the CI will determine whether the issue requires TMG or TSC action; (iii) any action taken and its rationale will be documented in the governance log (S4.7); (iv) the process evaluation team will consider the action as a contextual variable when interpreting subsequent data from the affected site(s).

## S4.6 Roles and Responsibilities

- **Process Evaluation Lead (GC):** Responsible for maintaining the boundary between evaluation and facilitation; reviews all proposed communications to the TMG for compliance with this protocol; leads the exception pathway where required.
- **Chief Investigator (TPH): Receives** exception pathway notifications; determines whether TMG or TSC action is warranted; authorises any deviation from restricted information sharing.
- **Trial Management Group:** Receives aggregate process evaluation indicators at scheduled meetings; does not receive site-specific qualitative data or interpretive analyses during the trial.
- **Trial Steering Committee:** Provides independent oversight of the governance protocol; reviews the governance log at scheduled TSC meetings; adjudicates any disputes about the evaluation-facilitation boundary.
- **Process Evaluation Researchers (JBS and field researchers):** Conduct data collection; do not provide implementation advice, coaching, or feedback to site staff during visits; refer site-level queries about the intervention protocol to the Trial Manager.

## S4.7 Documentation

A governance log will be maintained throughout the trial, recording: (i) all process evaluation communications to the TMG (date, content, format); (ii) any implementation support actions delivered through standard trial monitoring that were prompted by process evaluation observations; (iii) exception pathway activations (trigger, decision, action taken, rationale); (iv) any deviations from this protocol (with justification). The governance log will be reviewed by the TSC at each scheduled meeting and will be reported alongside process evaluation findings to enable readers to assess whether the evaluation-facilitation boundary was maintained.

## S4.8 Review and Amendment

This governance protocol will be reviewed at the end of the internal pilot phase (Month 12) and may be amended by agreement between the Process Evaluation Lead, Chief Investigator, and TSC. Any amendments will be version-controlled and documented in the governance log.
