# Supplementary material for: Randomised controlled trial of SHORT duration antibiotic thERapy for critically ill patients with sepsis (SHORTER trial): protocol for a mixed-methods process evaluation
Source: BMJ Open. 2026 Nov 2;16(11):e119173. doi: 10.1136/bmjopen-2026-119173 (PMC13735336; doi:10.1136/bmjopen-2026-119173)
Supplement: Supplementary file 7 [file bmjopen-16-11-s006.docx]

# Online Supplemental File 6

*A Randomised Controlled Trial of SHORT Duration Antibiotic thERapy for Critically Ill Patients With Sepsis (SHORTER Trial): Protocol for a Mixed-Methods Process Evaluation*

**Online supplemental Table S4. Example Interview Questions Mapped to Theoretical Constructs (Phase 1)**

| **Theoretical Level** | **Construct** | **Example Questions** |
| --- | --- | --- |
| **Micro (TPB)** | Attitudes | "What do you see as the main benefits of stopping antibiotics at day 5 for sepsis patients?" / "What concerns, if any, do you have about shorter antibiotic courses in this population?" |
|  | Subjective norms | "How do your colleagues generally view short-course antibiotic strategies?" / "Are there particular individuals whose opinions about antibiotic duration carry weight in your unit?" |
|  | Perceived behavioural control | "How confident do you feel about stopping antibiotics at day 5 when a patient is still unwell?" / "What would make it easier or harder for you to follow the 5-day protocol?" |
|  | Intention/Equipoise | "Under what circumstances might you feel unable to follow the protocol allocation?" / "How comfortable are you with genuine uncertainty about which treatment duration is better?" |
| **Meso (NPT)** | Coherence | "How would you explain the purpose of the 5-day strategy to a colleague unfamiliar with the trial?" / "How does this approach differ from your usual practice?" |
|  | Cognitive participation | "Who in your team has taken a lead role in implementing the trial?" / "Are there staff members who have expressed reservations about participating?" |
|  | Collective action | "How has the trial changed what happens during your ward rounds?" / "What new documentation or communication practices have you adopted?" |
|  | Reflexive monitoring | "How do you and your colleagues talk about how the trial is going?" / "Have you noticed any early effects—positive or negative—from participating?" |
| **Macro (CFIR)** | Inner setting | "How would you describe the culture around antibiotic prescribing in your unit?" / "What stewardship resources or support do you have access to?" |
|  | Outer setting | "How do external pressures—such as antimicrobial resistance targets or national guidelines—influence antibiotic decisions here?" |
|  | Process | "How was the decision made for your unit to participate in this trial?" / "What training or preparation did staff receive?" |

**Online supplemental Table S5. Composite Site Profile Scores Derived from Baseline Questionnaire**

| **Domain** | **Components** | **Score Range** | **Interpretation** |
| --- | --- | --- | --- |
| **Antimicrobial Stewardship Infrastructure** | Dedicated ICU pharmacist (Y/N); pharmacist time allocated to ICU (%); antibiotic prescribing protocol/policy in place (Y/N); structured de-escalation approach (protocol/bundle vs individualised) | 0-10 | Higher = more developed stewardship infrastructure |
| **Research Infrastructure** | Research nurse staffing (number, full/part-time, ICU experience); extent of recruitment cover (weekday daytime / 7-day daytime / 24-7); dedicated clinical trials pharmacist (Y/N); pharmacist active role in research (Y/N) | 0-10 | Higher = more developed research capacity |
| **Unit Complexity** | Staffed bed numbers (tertiles); level 2/3 case mix; annual admission rate; unit speciality | 0-10 | Higher = greater complexity |
| **Implementation Distance** | Unit consensus on standard antibiotic duration (10 days / 7 days / individualised); antibiotic prescribing/de-escalation protocol in place (Y/N); unit consensus on de-escalation (continue full course vs stop on negative cultures) | 0-10 | Higher = greater change required to achieve 5-day target |

Note: All items are drawn from the baseline site questionnaire (Supplement S5). Scores are equally weighted item sums, normalised to 0-10, and used for purposive sampling and cross-site comparison only; not intended for statistical prediction. Free-text questionnaire items (anticipated implementation challenges; perceived equipoise) are coded qualitatively to contextualise scores and are not scored.
