# Supplementary material for: Randomised controlled trial of SHORT duration antibiotic thERapy for critically ill patients with sepsis (SHORTER trial): protocol for a mixed-methods process evaluation
Source: BMJ Open. 2026 Nov 2;16(11):e119173. doi: 10.1136/bmjopen-2026-119173 (PMC13735336; doi:10.1136/bmjopen-2026-119173)
Supplement: Supplementary file 8 [file bmjopen-16-11-s007.docx]

**SPIRIT 2025 Checklist: Items to address in a clinical trial protocol**

Protocol title: A Randomised Controlled Trial of SHORT Duration Antibiotic thERapy for Critically Ill Patients With Sepsis (SHORTER Trial): Protocol for a Mixed-Methods Process Evaluation

Checklist per Chan AW, Boutron I, Hopewell S, et al. SPIRIT 2025 statement: updated guideline for protocols of randomised trials. BMJ 2025;389:e081477. doi: 10.1136/bmj-2024-081477

Note: This checklist has been adapted for a process evaluation protocol embedded within a clinical trial. Items concerning randomisation, blinding, clinical harms and post-trial care pertain to the host SHORTER trial (ISRCTN40090372) and are cross-referenced to the trial protocol rather than duplicated; adaptations are annotated below.

| **Section/topic** | **Item** | **SPIRIT 2025 item description** | **Addressed** | **Location in manuscript** |
| --- | --- | --- | --- | --- |
| **ADMINISTRATIVE INFORMATION** |  |  |  |  |
| Title and structured summary | 1a | Title stating the trial design, population, and interventions, with identification as a protocol | Yes | Title (title page; p.1) |
|  | 1b | Structured summary of trial design and methods, including items from the WHO Trial Registration Data Set | Yes | Abstract; title page |
| Protocol version | 2 | Version date and identifier | Partial | Title and title page identify this as a protocol; a separate version identifier/date for the process-evaluation protocol is not stated. Section 6 reports that the published host-trial manuscript is based on protocol V6.0 (24 September 2025). |
| Roles and responsibilities | 3a | Names, affiliations, and roles of protocol contributors | Yes | Title page; Section 9 |
|  | 3b | Name and contact information for the trial sponsor | Partial | Section 5 (sponsor name); host trial protocol, Key Trial Contacts (sponsor contact). Sponsor contact information is not reproduced in this manuscript. |
|  | 3c | Role of trial sponsor and funders in design, conduct, analysis, and reporting of trial; including any authority over these activities | Yes | Section 7; title page (funding note) |
|  | 3d | Composition, roles, and responsibilities of the coordinating site, steering committee, endpoint adjudication committee, data management team, and other individuals or groups overseeing the trial, if applicable | Yes, adapted | Section 4.1; Table 2; Supplement S4, especially S4.5–S4.7 (CI, TMG, TSC, process-evaluation lead and researchers); host trial protocol §10.3. |
| **OPEN SCIENCE** |  |  |  |  |
| Trial registration | 4 | Name of trial registry, identifying number (with URL), and date of registration. If not yet registered, name of intended registry | Yes | Title page; Section 6 (registered 12 Dec 2023) |
| Protocol and statistical analysis plan | 5 | Where the trial protocol and statistical analysis plan can be accessed | Yes | Published host trial protocol (Mawson et al., 2026; ISRCTN40090372); analysis plan: Supplement S1 |
| Data sharing | 6 | Where and how the individual de-identified participant data (including data dictionary), statistical code, and any other materials will be accessible | Partial | Section 10 states the materials intended for release and controlled access, but does not yet specify a repository, persistent location or controlled-request route. |
| Funding and conflicts of interest | 7a | Sources of funding and other support (e.g., supply of drugs) | Yes | Section 7 |
|  | 7b | Financial and other conflicts of interest for principal investigators and steering committee members | Partial | Section 8 covers manuscript authors. Competing-interest declarations for all principal investigators and independent steering-group members are not stated in this process-evaluation protocol. |
| Dissemination policy | 8 | Plans to communicate trial results to participants, healthcare professionals, the public, and other relevant groups (e.g., reporting in trial registry, plain language summary, publication) | Partial | Section 5 covers peer-reviewed publication, conferences, accessible patient/public summaries and clinical/policy networks; direct participant communication and trial-registry results reporting are not explicit. |
| **INTRODUCTION** |  |  |  |  |
| Background and rationale | 9a | Scientific background and rationale, including summary of relevant studies (published and unpublished) examining benefits and harms for each intervention | Yes | Section 1 |
|  | 9b | Explanation for choice of comparator | Yes | Section 1 (host trial summary) |
| Objectives | 10 | Specific objectives related to benefits and harms | Yes, adapted | Section 2 (process evaluation objectives) |
| **METHODS: PPI AND TRIAL DESIGN** |  |  |  |  |
| Patient and public involvement | 11 | Details of, or plans for, patient or public involvement in the design, conduct, and reporting of the trial | Yes | Section 4.7 (Patient and Public Involvement) |
| Trial design | 12 | Description of trial design including type of trial (e.g., parallel group, crossover), allocation ratio, and framework (e.g., superiority, equivalence, non-inferiority, exploratory) | Yes | Section 1 and Section 4.1; host trial protocol §7.2 (parallel, 1:1 randomisation); host trial is pragmatic, open-label and non-inferiority for mortality. |
| **METHODS: PARTICIPANTS, INTERVENTIONS, AND OUTCOMES** |  |  |  |  |
| Trial setting | 13 | Settings (e.g., community, hospital) and locations (e.g., countries, sites) where the trial will be conducted | Yes | Section 1; Section 4.2.1 |
| Eligibility criteria | 14a | Eligibility criteria for participants | Yes, adapted | Section 4.2.2 (eligible staff roles at participating sites); Section 1 (host trial patients) |
|  | 14b | If applicable, eligibility criteria for sites and for individuals who will deliver the interventions (e.g., surgeons, physiotherapists) | Yes | Section 4.2.1 (all participating trial sites eligible; 10 sampled by maximum variation) |
| Intervention and comparator | 15a | Intervention and comparator with sufficient details to allow replication including how, when, and by whom they will be administered. If relevant, where additional materials describing the intervention and comparator (e.g., intervention manual) can be accessed | Yes, adapted | Section 1 (host trial); Sections 4.3.1–4.3.4 (process evaluation procedures) |
|  | 15b | Criteria for discontinuing or modifying allocated intervention/comparator for a trial participant | Yes — host trial; adapted | Host trial protocol (Mawson et al., 2026; ISRCTN40090372), §§7.7 and 8.3–8.4; exception/non-adherence criteria summarised in Section 4.4.2 and Table 4. |
|  | 15c | Strategies to improve adherence to intervention/comparator protocols, if applicable, and any procedures for monitoring adherence | Yes, adapted | Section 4.1 (support log); Section 4.4.2 (fidelity monitoring) |
|  | 15d | Concomitant care that is permitted or prohibited during the trial | Yes — host trial | Host trial protocol (Mawson et al., 2026; ISRCTN40090372), §§8.1–8.2 (antibiotic choice, additional antibiotics, biomarkers and usual concomitant care). |
| Outcomes | 16 | Primary and secondary outcomes, including the specific measurement variable, analysis metric, method of aggregation, and time point for each outcome | Yes, adapted | Section 2; Section 4.4.2; Table 4 (process outcomes: fidelity, separation, variation) |
| Harms | 17 | How harms are defined and will be assessed (e.g., systematically, non-systematically) | Yes — host trial; adapted | Host trial protocol (Mawson et al., 2026; ISRCTN40090372), §§10.1–10.4 (definitions, collection, reporting and oversight); process-evaluation observation safeguards: Supplement S2, S2.5. |
| Participant timeline | 18 | Time schedule of enrolment, interventions (including any run-ins and washouts), assessments, and visits for participants. A schematic diagram is highly recommended | Yes | Figure 2 and Table 1 (process-evaluation schedule); host trial protocol §7.6.1 (participant schedule of events). |
| Sample size | 19 | How sample size was determined, including all assumptions supporting the sample size calculation | Yes, adapted | Sections 4.2.1–4.2.2 (information power) |
| Recruitment | 20 | Strategies for achieving adequate participant enrolment to reach target sample size | Yes | Sections 4.2.2 and 4.3.3 (recruitment across professional groups; PI nomination and snowball sampling for less-engaged clinicians) |
| **METHODS: ASSIGNMENT OF INTERVENTIONS** |  |  |  |  |
| Randomisation: sequence generation | 21a | Who will generate the random allocation sequence and the method used | N/A — host trial | Host trial protocol (Mawson et al., 2026; ISRCTN40090372); process evaluation does not randomise participants |
|  | 21b | Type of randomisation (simple or restricted) and details of any factors for stratification | N/A — host trial | Host trial protocol (Mawson et al., 2026; ISRCTN40090372) |
| Allocation concealment mechanism | 22 | Mechanism used to implement the random allocation sequence, describing any steps to conceal the sequence until interventions are assigned | N/A — host trial | Host trial protocol (Mawson et al., 2026; ISRCTN40090372) |
| Implementation | 23 | Whether the personnel who will enrol and those who will assign participants to the interventions will have access to the random allocation sequence | N/A — host trial | Host trial protocol (Mawson et al., 2026; ISRCTN40090372) |
| Blinding | 24a | Who will be blinded after assignment to interventions (e.g., participants, care providers, outcome assessors, data analysts) | Adapted | Host trial is open-label; fidelity coding conducted blind to site identity and clinical outcomes (Section 4.4.2) |
|  | 24b | If blinded, how blinding will be achieved and description of the similarity of interventions | N/A | — |
|  | 24c | If blinded, circumstances under which unblinding is permissible, and procedure for revealing an allocated intervention during the trial | N/A | — |
| **METHODS: DATA COLLECTION, MANAGEMENT, AND ANALYSIS** |  |  |  |  |
| Data collection methods | 25a | Plans for assessment and collection of trial data, including processes to promote data quality and a description of trial instruments along with their reliability and validity, if known. Reference to where data collection forms can be accessed, if not in the protocol | Yes | Section 4.3.1 (instrument development and validation); Sections 4.3.2–4.3.4; Table 3; online supplemental Tables S4–S5; Supplements S3, S5 |
|  | 25b | Plans to promote participant retention and complete follow-up, including list of any outcome data to be collected for participants who discontinue or deviate from intervention protocols | Partial, adapted | Sections 4.2.2 and 4.3.4 (voluntary longitudinal re-interviews); host trial protocol §7.7 (withdrawal and retained follow-up data). No additional staff-participant retention procedure is specified. |
| Data management | 26 | Plans for data entry, coding, security, and storage, including processes to promote data quality. Reference to where details of data management procedures can be accessed, if not in the protocol | Yes | Section 4.5; Supplement S1, S1.3 (qualitative coding quality assurance), S1.4.3 (routine-data completeness/missingness) and S1.6 (version-controlled analysis and deviation log); Supplement S2, S2.6. |
| Statistical methods | 27a | Statistical methods used to compare groups for primary and secondary outcomes, including harms | Yes, adapted | Section 4.4.2 |
|  | 27b | Definition of who will be included in each analysis (e.g., all randomised participants), and in which group | Yes | Section 4.4.2 (ITT and evaluable denominators; NoMAD inclusion thresholds) |
|  | 27c | How missing data will be handled in the analysis | Yes | Section 4.4.2; Supplement S1 (S1.4.2 inclusion hierarchy; S1.4.3 routine-data missing-data rules; Table S3 scoring and missing-item rules) |
|  | 27d | Methods for any additional analyses (e.g., subgroup and sensitivity analyses) | Yes | Section 4.4.2 (exploratory site-level analyses); Section 4.4.3 (sensitivity analyses of cut points; infection-type subgroups) |
| **METHODS: MONITORING** |  |  |  |  |
| Data monitoring committee | 28a | Composition of data monitoring committee (DMC); summary of its role and reporting structure; statement of whether it is independent from the sponsor and funder; conflicts of interest; where its charter can be found. Alternatively, an explanation of why a DMC is not needed | Partial — host trial | Host trial protocol (Mawson et al., 2026; ISRCTN40090372), §10.3.5 (DMC safety-review role); process-evaluation oversight: Section 4.1 and Supplement S4. DMC composition, independence, conflicts and charter location are not stated here. |
|  | 28b | Explanation of any interim analyses and stopping guidelines, including who will have access to interim results and make the final decision to terminate the trial | Yes — host trial; adapted | Host trial protocol (Mawson et al., 2026; ISRCTN40090372), §§7.9, 10.3.5 and 11.2.5 (DMC review, no formal interim analysis/predefined stopping rules, early-stop authority); process-evaluation feedback: Section 4.1, Table 2 and Supplement S4. |
| Trial monitoring | 29 | Frequency and procedures for monitoring trial conduct. If there is no monitoring, give explanation | Yes | Section 4.1 (support log); Section 4.4.2 (monthly delivery metrics) |
| **ETHICS** |  |  |  |  |
| Research ethics approval | 30 | Plans for seeking research ethics committee/institutional review board approval | Yes | Section 5 |
| Protocol amendments | 31 | Plans for communicating important protocol modifications to relevant parties | Partial, adapted | Supplement S4, S4.8 (governance-protocol review/amendment); Supplement S1, S1.6 (analysis-plan deviations); host trial protocol §16.9 (substantial amendments). A separate process-evaluation protocol-modification communication route is not stated. |
| Consent or assent | 32a | Who will obtain informed consent or assent from potential trial participants or authorised proxies, and how | Partial | Section 5 and Supplement S2, S2.2/S2.4 and approved consent forms describe how consent is documented; the staff role authorised to obtain process-evaluation consent is not explicitly specified. |
|  | 32b | Additional consent provisions for collection and use of participant data and biological specimens in ancillary studies, if applicable | N/A | No ancillary collection or use of biological specimens is planned for the process evaluation; host-trial provisions are in the host trial protocol (Mawson et al., 2026; ISRCTN40090372). |
| Confidentiality | 33 | How personal information about potential and enrolled participants will be collected, shared, and maintained in order to protect confidentiality before, during, and after the trial | Yes | Section 4.5; Section 5 |
| Ancillary and post-trial care | 34 | Provisions, if any, for ancillary and post-trial care, and for compensation to those who suffer harm from trial participation | N/A | Staff-participant evaluation; no post-trial care applicable. Host trial: trial protocol |
